# Integrating Enzyme-Based Kinetics in Reactive Transport Models to Simulate Spatiotemporal Dynamics of Biomarkers during Chlorinated Ethene Degradation

**DOI:** 10.1021/acs.est.4c07445

**Published:** 2024-11-07

**Authors:** Diego Di Curzio, Michele Laureni, Mette M. Broholm, David G. Weissbrodt, Boris M. van Breukelen

**Affiliations:** † Department of Water Management, Delft University of Technology, Stevinweg 1, 2628 CN Delft, Netherlands; ‡ Department of Environmental and Resource Engineering, Technical University of Denmark, Bygningstorvet 115, 2800 Kongens Lyngby, Denmark; § Department of Biotechnology and Food Science, Norwegian University of Science and Technology, Sem Sælandsvei 8, 7034 Trondheim, Norway

**Keywords:** biodegradation, organohalide respiration, reductive dechlorination, enzyme-based kinetics, reactive transport modeling, contaminated groundwater

## Abstract

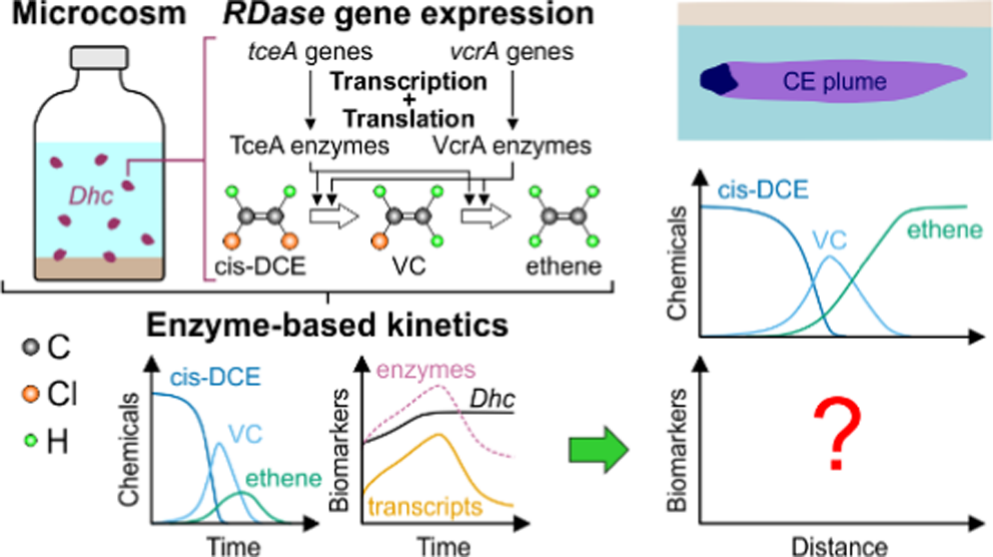

Biomarkers such as functional gene mRNA (transcripts) and proteins (enzymes) provide direct proof of metabolic regulation during the reductive dechlorination (RD) of chlorinated ethenes (CEs). Yet, current models to simulate their spatiotemporal variability are not flexible enough to mimic the homologous behavior of *RDase* functional genes. To this end, we developed new enzyme-based kinetics to model the concentrations of CEs together with the transcript and enzyme levels during RD. First, the model was calibrated to existing microcosm data on RD of cis-DCE. The model mirrored the *tceA* and *vcrA* gene expression and the production of their enzymes in *Dehalococcoides* spp. Considering *tceA* and *vcrA* as homologous instead of nonhomologous improved fitting of the mRNA time series. Second, CEs and biomarker patterns were explored as a proof of concept under groundwater flow conditions, considering degraders occurring in immobile and mobile states. Under both microcosm and flow conditions, biomarker-rate relationships were nonlinear hysteretic because *tceA* and *vcrA* acted as homologous genes. The mobile biomarkers additionally undergo advective-dispersive transport, which increases the nonlinearity and makes the observed patterns even more challenging to interpret. The model offers a thorough mechanistic description of RD while also allowing simulation of spatiotemporal dynamic patterns of various key biomarkers in aquifers.

## Abstract 2

Organohalide-respiring bacteria synthesize biomarkers while degrading chlorinated solvents. This paper introduces a new approach to interpret these biomarker spatiotemporal patterns, advancing our understanding of biodegradation dynamics in contaminated aquifers.

## Introduction

Chlorinated ethenes (CEs) are highly toxic and carcinogenic chemicals known to negatively impact human health and ecosystems.^1^ Their degradation occurs mostly through microbially catalyzed redox reactions referred to as organohalide respiration^2^ and known as reductive dechlorination^3,4^ (RD). Organohalide-respiring bacteria (OHRB) use CEs as electron acceptors for ATP generation and growth.^5^ They reduce CEs by converting organics or dihydrogens to electron donors.

The use of biomarkers (i.e., 16S rRNA gene and functional gene DNA, mRNA, and proteins) to investigate metabolic functions of OHRB^6−10^ and infer RD rates in microcosms^11−16^ and column experiments^17^ is well established. Since these biomarkers contribute to better monitoring of biotic RD occurrence^18−21^ and owing to optimized sampling protocols ensuring reliable quantification, they are also increasingly being measured in polluted site investigations, e.g., to assess the occurrence of natural attenuation^22−25^ and to monitor the efficiency of bioaugmentation or biostimulation interventions.^26−29^ However, the ongoing challenge lies in developing a model that can simulate the spatiotemporal dynamics of biomarkers in polluted aquifers to enhance the mechanistic understanding of microbially driven RD, more reliable rate predictions, and overall better constrained RD simulations.

Reactive transport models (RTMs) are state-of-the-art process-based tools that can simulate the evolution of biodegradation of CEs in aquifers under natural conditions^30−32^ and during bioremediation.^33−37^ RTMs provide spatiotemporal CE concentration patterns, including their compound-specific stable isotope ratios,^38−40^ yet the explicit integration of biomarkers remains very limited. To date, time-dependent RD has been modeled through kinetics,^41^ and only in some cases, biomarkers have been explicitly included as constraints. The total amount of OHRB, estimated by 16S rRNA gene quantification, has been successfully used to constrain either Michaelis–Menten^42,43^ or Monod-type kinetics.^29,44−47^ In Rowe et al.,^14^ chloroethene respiration rates of *Dehalococcoides* were related to enzyme levels by including the average protein abundances in a Michaelis–Menten equation. Heavner et al.^48^ used mRNA to adjust the activities of individual bacterial populations in their Monod-like kinetics to model the competition for H_2_ as an electron donor between *Dehalococcoides* and hydrogenotrophic methanogens. The use of biomarkers in these papers has resulted in improved predictions. However, none of these models has explicitly incorporated the metabolic functions of OHRB, which would make biodegradation rates physically based and more reliable.

Bælum et al.^49^ developed an enzyme-based approach able to link RD of vinyl chloride (VC) to the expression of a specific functional gene (i.e., VC reductive dehalogenase, *vcrA*) in *Dehalococcoides* in the KB1 culture. The model explicitly simulated the metabolic regulatory chain of *vcrA* expression including transcription factors, mRNA, enzymes, and bacterial growth. However, plumes contain more than one CE, and the expression of a single *RDase* functional gene can contribute to multiple steps of sequential RD,^9,24,50−54^ in fact acting as homologous genes.^55^ At a community level, multiple functional genes can be simultaneously expressed by OHRB to produce specific RDase enzymes.^16^

Enzyme-based kinetics have been implemented to model more complex metabolic regulation dynamics in sequential denitrification using the well-known metabolic regulatory system of *Paracoccus denitrificans*.^56^ Störiko et al.^56^ were the first to simulate the activation of transcription factors by both nitrate and nitrite and their inhibition by O_2_. However, each denitrification step was driven by one type of enzyme, as in Bælum et al.^49^ Furthermore, the biomass growth was not linked to denitrification but instead depended on an external electron acceptor (i.e., O_2_).

In this study, we aim to develop a novel approach to model spatiotemporal patterns of the biomarkers linked to organohalide respiration in *Dehalococcoides*. To this end, we implemented novel enzyme-based kinetics to model the regulation of CE biodegradation through *RDase* gene expression and enzyme production. The model introduces key features that were never incorporated in previous attempts,^49,56^ making it more flexible: (i) the presence of homologous functional genes expressed by different *Dehalococcoides* strains at a community level, (ii) nonunique transcription regulation mechanisms in CE-respiring OHRB,^57^ and (iii) *Dehalococcoides* explicitly growing on multiple CEs. First, we calibrated the model to batch experimental data gained from the literature^58^ and compared the results with the well-known Monod kinetics to prove our model as reliable for simulating biotic RD and biomass growth; subsequently, we applied the model in 1D to simulate the development of a cis-DCE plume in groundwater and considering *Dehalococcoides* being distributed between the solid and aqueous phase. In this way, we provided spatiotemporal patterns of CEs, biomarkers, degradation rates expected under field conditions and mechanistic insights regarding the biogeochemical processes underlying RD. This modeling endeavor provides for the first time a systematic modeling framework to understand and localize transformation processes of organohalides in groundwater.

## Material and Methods

### Data Set Description

Measurements of chemicals (i.e., cis-DCE, VC, ethene, and chloride) and biomarkers (i.e., 16S rRNA gene of *Dehalococcoides* spp., and *tceA* and *vcrA* functional gene transcripts) from Kranzioch et al.^58^ were used for model conceptualization and calibration. In that study, microcosm experiments were performed to evaluate the dechlorination capability of sediments from the Yangtze River (China) and to investigate *RDase* gene expression. Among these experiments, we selected the data from the cis-DCE-spiked culture, where the complete dechlorination to ethene was linked to the *Dehalococcoides* spp. (just *Dehalococcoides* later on) respiration. Additional details are provided in Section S1.

### Metabolic Pathways and Microcosm Conditions

**Figure 1:**
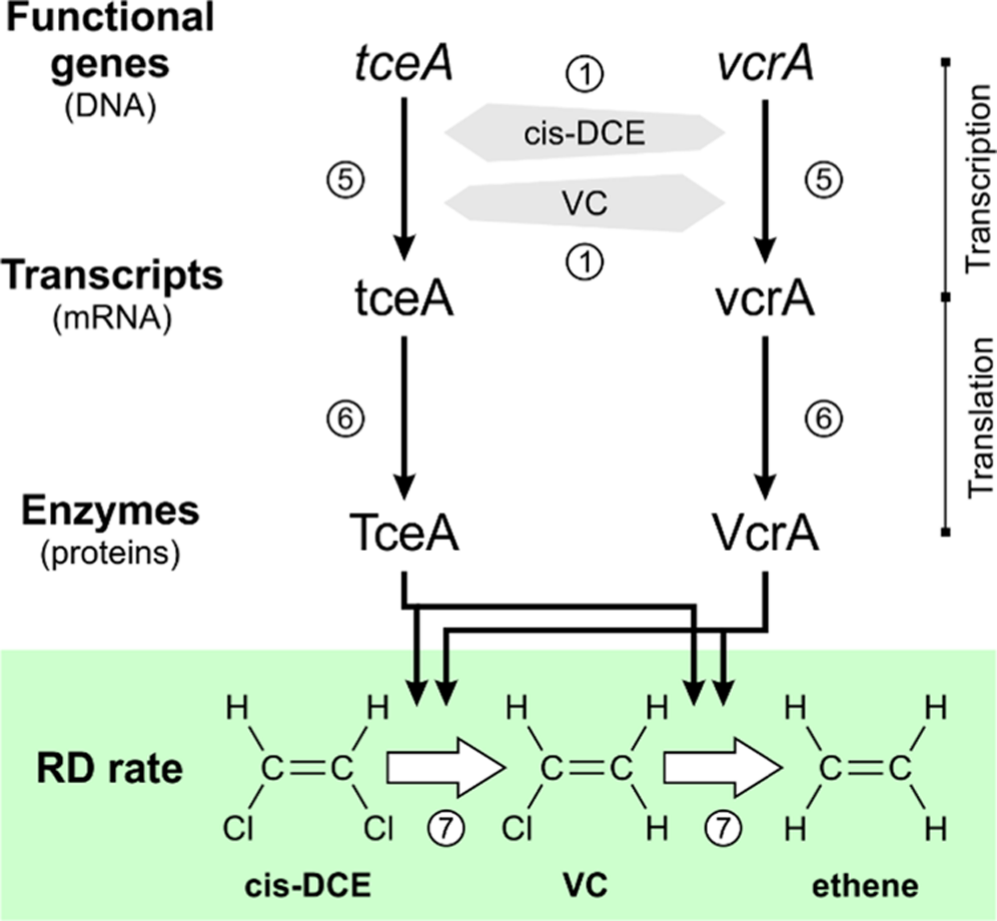
Conceptual model of the metabolic pathways responsible for the complete biodegradation of cis-1,2-DCE to ethene in the microcosm experiment from Kranzioch et al.^58^ considered in this study. The light gray polygons represent the concentration-dependent activation of the generic transcription factors by cis-1,2-DCE and VC. Numbers in the white circles refer to the equations described in the following section, representing specific reactions of the considered metabolic pathways.

In the conceptual model (Figure 1), *Dehalococcoides* transcribe the reductive dehalogenase genes *tceA* (i.e., trichloroethene reductive dehalogenase) and *vcrA* (i.e., vinyl chloride reductive dehalogenase) into mRNA, which is subsequently translated into proteins and activated into the TceA and VcrA enzymes. TceA and VcrA catalyze the degradation of cis-DCE and VC and determine the corresponding transformation rates.^24,54^ The transcription regulation mechanisms in chloroethene-respiring OHRB are diverse and remain partially unresolved. Depending on the species, transcription may be regulated via a two-component system, *MarR*-type regulators, or CPR/FNR-type regulators,^57^ and the transcription mechanism itself depends on the presence of different substrates.^59^ In our work, we attributed the transcription regulation to a generic rather than specific transcription regulation system with both cis-DCE and VC as activators of both *tceA* and *vcrA* expression. Even though the *tce*-carrying *Dehalococcoides* are often considered to contribute to the VC-to-ethene RD step co-metabolically,^60^ it has been observed that both *tceA*- and *vcrA*-carrying strains can degrade both cis-DCE and VC metabolically.^9,24^ Accordingly, we assumed that *Dehalococcoides* grow on both the cis-DCE and VC.

### Reductive Dechlorination Kinetics Implementation and Model Calibration

We developed an enzyme-based kinetic model of the metabolic pathways responsible for the complete biodegradation of cis-DCE to ethene (Figure 1). In this approach, the activation rate of generic transcription factors (*r*_Xact_^i^ [s^–1^]) is described by eq 1:
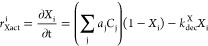
1where *C*_j_ [mol·CE·L^–1^] and *a*_j_ [L·mol·CE^–1^·s^–1^] are the total concentration and the activation rate coefficient of each CE promoting each transcription factor (*X*_i_ [-]) activation, respectively, and *k*_dec_^X^ [s^–1^] is the transcription factor dissociation constant. As in Störiko et al.,^56^ the term (1 – *X*_i_) [-] was introduced to consider the inactive fraction of the *i*th transcription factors still available to bind to the CEs activating the functional gene expression, since there is a finite quantity of them in cells.

The rate of functional gene transcription (*r*_tsc_^i^ [mol transcripts L^–1^·s^–1^]) is defined in eq 2:
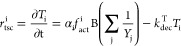
2where *T*_i_ [mol·transcript·L^–1^] represent the transcript amount and α_i_ [mol·transcripts·mol·biomass^–1^·s^–1^] the maximum transcription rate of each functional gene i; *k*_dec_^T^ [s^–1^] is the first-order coefficient describing transcript decay, whereas *Y*_j_ [mol of biomass mol of substrate^–1^] is the yield factor corresponding to the *j*th CE promoting transcription; and B [mol of biomass L^–1^] is the concentration of *Dehalococcoides* in the solution. The fraction of active operator sites (*f*_act_^i^ [-]) is linked to the transcription factor activation using a Hill function:^61^
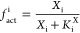
3with *K*_i_^X^ [-] being the half-saturation constant of transcription factor binding to the operator sites.

The enzymes responsible for the dechlorination of CEs (*E*_i_ [mol·enzymes·L^–1^]) are produced via translation once functional genes have been transcribed, as follows:
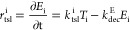
4where *r*_tsl_^i^ [mol·enzymes·L^–1^·s^–1^] is the translation rate, *k*_tsl_^i^ [mol·enzymes·mol·transcript^–1^·s^–1^] is the first-order translation coefficient, and *k*_dec_^E^ [s^–1^] is the unique first-order enzyme decay coefficient.

Transcription and translation occur quickly, usually within minutes or hours. In contrast, reductive dechlorination can take tens of days or months because of diverse limiting factors (e.g., electron donor availability), even under controlled conditions like microcosms (e.g., Michalsen et al.^16^). Therefore, differently than Bælum et al.^49^ and Störiko et al.^56^ who modeled at least one step between transcription and translation because the simulated complete degradation occurred very rapidly (i.e., 10 days and 45 h, respectively, while >40 days in our case), we simulated both transcript and enzyme concentrations at quasi-steady-state conditions in the case of organohalide respiration by assuming  and . This simplification allowed us to define quasi-steady-state transcript (*T*_i_^qss^) and enzyme (*E*_i_^qss^) levels as follows:
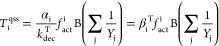
5
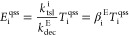
6where β_i_^T^ [mol·transcripts·mol·biomass^–1^] and β_i_^E^ [mol·enzymes·mol·transcripts^–1^] are the maximum concentration of transcripts per mol of biomass and of enzymes produced per mole of transcripts for each functional gene i. Under quasi-steady-state conditions, transcript and enzyme levels are linearly proportional, and both the transcription factors and the active biomass control their variation over time. With this assumption, the *Dehalococcoides* metabolism and growth are predominantly regulated at the transcriptional level.^62^

Once the amount of enzyme is defined at each time step, we calculated the RD rate (*r*_RD_^j^ [mol·L^–1^·s^–1^]) via eq 7:
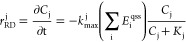
7where *k*_max_^j^ [mol·CE·mol·enzymes^–1^·s^–1^] is the maximum amount of CE that can degrade by a mole of enzymes per unit of time, while *K*_j_ [mol·L^–1^] is the half-saturation constant of the jth CE.

*Dehalococcoides* growth (*r*_grt_ [mol·biomass·L^–1^·s^–1^]) is modeled using the following equation:
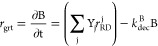
8where *k*_dec_^B^ [s^–1^] is the first-order coefficient for bacterial decay.

RD of CEs was also modeled through traditional Monod kinetics for comparison by eq 9:
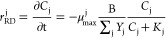
9where μ_max_^j^ [s^–1^] is the specific bacterial growth rate, which links the RD rate to the bacterial growth rate described by eq 8.

Ethene degradation was modeled following first-order kinetics because it generally occurs via different reaction pathways^63^ and is not related to organohalide respiration in *Dehalococcoides*.

In the equations, biomass is considered as [mol·L^–1^] of carbon equivalent to maintain the consistency of units, as the yield factor is equal to [mol of biomass mol of CEs^–1^]. Since each bacterial cell contains approximately 10^–14^ moles of carbon^64^ and both transcripts and enzymes are proportional to the *Dehalococcoides* concentration, we used the same proportionality value (Table S1) to convert the biomarker concentrations in [mol·L^–1^] to the corresponding number of units per liter (i.e., [genes-transcripts-enzymes L^–1^]).

Two batch models were implemented in PHREEQC v. 3.6.2,^65^ and kinetic parameters (Table S1) were estimated from experimental data via automatic model calibration performed with PEST v.17 using the Gauss–Marquardt–Levenberg method,^66^ as commonly done in similar studies.^67,68^ Further details about the model calibration are provided in Section S2.

### 1D Scenario Reactive Transport Modeling

We used the model to simulate spatial patterns of biomarkers in 1D flow conditions as a proof of concept of what could be expected in aquifers in a scenario describing the development of a cis-DCE plume from a contaminant source in two stages: when the plume is extending downstream from the source (i.e., the elongation stage, after 10 years) and when the plume has reached the steady-state condition (i.e., after 40 years).

The biodegradation of cis-DCE was modeled along a 1500 m long flowline (i.e., 60 cells; 25 m cells), considering a groundwater steady-state flow rate of 100 m/y (i.e., 0.25-y time steps). The aquifer dispersivity was set equal to 5 m, which aligns with experimental values from the literature for homogeneous aquifers.^69^ Continuous release of cis-DCE was simulated, with a constant concentration of 50 μmol/L (4.85 mg/L) in the source zone. Sorption and thus retardation of CEs and ethene were simulated assuming an organic carbon content of 0.5% (Section S3).

*Dehalococcoides* were considered growing on the solid matrix either as biofilm or attached cells (i.e., immobile *Dehalococcoides*) and partly being released in the porewater (i.e., mobile *Dehalococcoides*), as it has been observed that *Dehalococcoides* are predominantly associated with solid phase during RD.^70,71^ In this model, immobile *Dehalococcoides* represented 85% of the total, whereas the remaining 15% were considered mobile and undergoing advective-dispersive transport. Further details are given in Section S4.

The initial amount of *Dehalococcoides* on the solid matrix was set equal to 920 genes/kg (5700 genes/mass of soil containing 1 L of porewater), yielding 10^3^ genes/L (1 genes/mL) in the aqueous phase, which is a typical value for pristine groundwater.^25^ To make the simulation more realistic and include the effect of the available pore space in limiting the immobile microbial growth, the following logistic-type term was used to constrain eq 8 and prevent the unrealistic boundless growth, as proposed by Carrera et al.:^72^
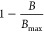
10where *B*_max_ is the maximum amount *Dehalococcoides* allowed in the pore space. This value was set equal to 2·10^7^ genes/kg (1.2·10^8^ genes/mass of soil containing 1 L of porewater), which aligns with values observed in plumes undergoing RD.^73^

Electron donor limitation was not considered in the flow models, as already observed in CE plumes.^25^ Furthermore, ethene conversion was not simulated, as under nonmethanogenic conditions.^63,74^

## Results and Discussion

### Biodegradation of Chlorinated Ethenes under Microcosm Conditions

**Figure 2:**
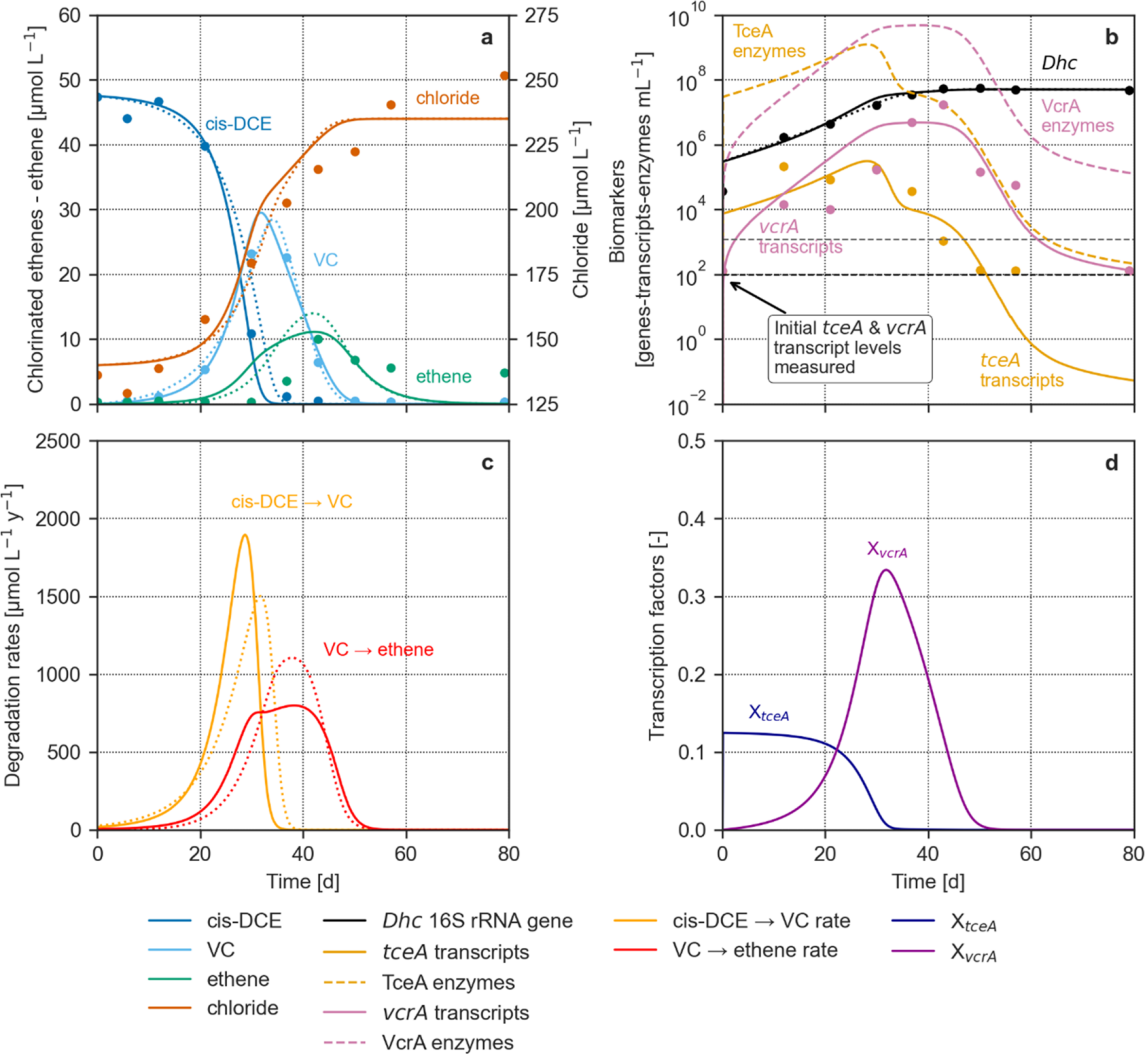
Temporal patterns of chemical concentrations (a), biomarker levels (b), degradation rates (c), and transcription factors (d) in the microcosm experiment of Kranzioch et al.,^58^ obtained through the enzyme-based kinetics and considering the metabolic pathways in Figure 1 (solid and dashed lines). The CE, ethene, and chloride concentrations, as well as the *Dehalococcoides* level and the degradation rates obtained through Monod kinetics, are shown as dotted lines. In the plots, the concentration of 16S rRNA gene copies corresponds to the number of *Dehalococcoides* cells. The black and gray dashed lines represent the quantification limits of the *Dehalococcoides* 16S rRNA gene and the *tceA* and *vcrA* transcripts, respectively. Refer to the original paper for the error bars related to the standard deviation of all of the chemical and biomarker measurements.

The enzyme-based kinetics (EBK) model linking the CEs biodegradation to *RDase* gene expression was fitted to batch experimental data and compared to the results obtained by a more traditional Monod approach (Figure 2).

The EBK model performed satisfactorily as the Monod model in simulating the complete biodegradation of cis-DCE to ethene (Figure 2a), capturing the marked increase in degradation rates (Figure 2c) driven by the exponential growth of *Dehalococcoides* on both cis-DCE and VC (Figure 2b), during the first 35 days. Upon complete depletion of cis-DCE, the models also captured the immediate decrease in the bacterial growth and degradation rates (Figure 2c), with VC as the sole electron acceptor. The resulting sequential degradation pattern is also well described by the measured and simulated chloride evolution, with a rapid rise until the complete cis-DCE disappearance, followed by a slower increase until VC and cis-DCE are completely dechlorinated. After the complete disappearance of CEs, transcripts and enzymes downregulated rapidly, while the *Dehalococcoides* concentration remained high, because bacteria were dying off at a slower pace. The decorrelation between the concentration of *Dehalococcoides* and the levels of both transcripts and enzymes suggests that bacteria temporarily deactivate before dying off. The organohalide respiration is crucial for active *Dehalococcoides*; thus, these bacteria can temporarily survive without expressing *RDase* genes but only in an inactive state.

By numerically linking bacterial growth to the metabolic regulation underlying organohalide respiration, the EBK model could also simulate the *tceA* and *vcrA* transcripts (Figure 2b). *tceA* transcripts increased rapidly at the beginning of the simulation and started decreasing slowly between day 30 and 45; *vcrA* transcripts also started being produced slower during the first 35 days, reaching the highest concentration between day 30 and 45. The transcripts’ temporal patterns suggest that the *tceA* gene was mainly expressed during cis-DCE dechlorination, while *vcrA* transcripts were upregulated when VC degradation was prevalent. However, the slow downregulation of *tceA* transcripts during the transformation of VC to ethene (i.e., between day 30 and 45) and the slow, gradual *vcrA* transcripts upregulation during the degradation of cis-DCE (i.e., between days 30 and 45) suggest that both electron acceptors activate the transcription of both functional genes (Figure 2d) as we defined in the proposed enzyme-based kinetics, even though influencing them differently (i.e., see values of *a*_j_ in eq (1) in Table S1). To emphasize the contribution of both homologous *RDase* genes to each degradation step, the results of a simulation considering nonhomologous *tceA* and *vcrA* gene expression (i.e., linked to a single CE, similarly to Bælum et al.^49^) are shown in Figure S1. As expected, RD is significantly less efficient than in the case of homologous *RDase* genes. The lack of cooperation between enzyme groups creates longer tails in the time series of cis-DCE and VC, which, in turn, keep both transcripts and enzymes overly upregulated. Moreover, the modeled peak values of *tceA* and *vcrA* transcripts were lower than in the case of homologous genes. This comparison proved the homologous gene assumption to be crucial for implementing our model.

Due to the quasi-steady-state assumption linearly linking transcripts and enzymes (eq 6), the abundances of the TceA and VcrA enzymes showed similar temporal behavior as their corresponding transcripts, with concentrations 3 orders of magnitude higher (Figure 2b). Since the translation process was unconstrained because of the lack of enzyme measurements in Kranzioch et al.,^58^ we used values (Table S1) of the maximum concentration of enzymes produced per mole of transcripts (i.e., β_i_^E^ in eq 6) in line with literature values.^75^ Despite the uncertainty resulting from the lack of constraining measurements, we could calculate enzyme concentrations consistent with experimental observations.^16^ This evidence further supports the validity of our model, which was implemented considering each degradation step dependent on both TceA and VcrA, as widely observed in the literature.^9,24,50−55^

### *RDase* Gene Expression in Contaminant Plumes

**Figure 3:**
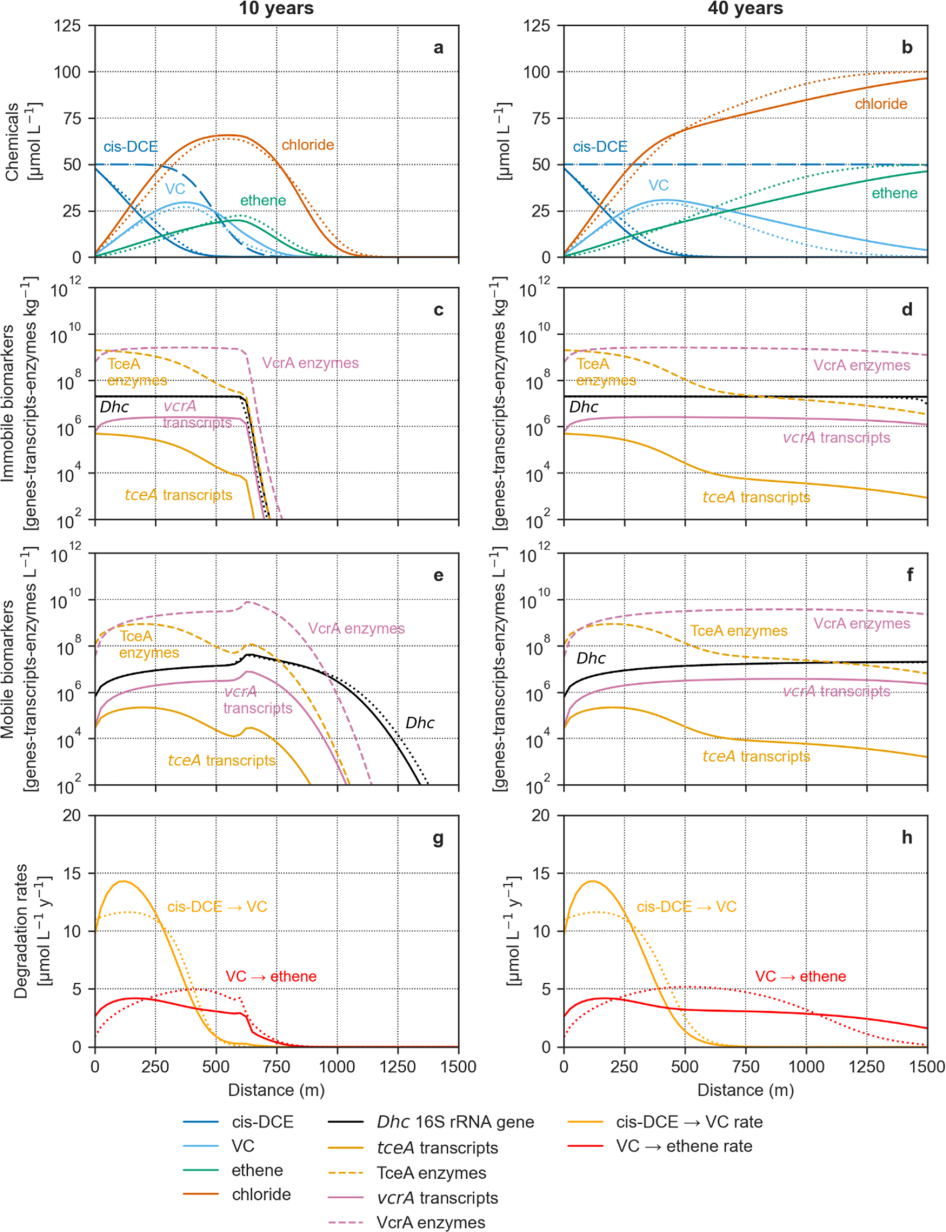
1D scenario-based reactive transport model simulating two relevant stages of the evolution of a cis-DCE plume in groundwater: the elongation phase at 10 years, when the plume is in a transient state, and the steady-state condition at 40 years. In addition to chemicals (a, b), the plot includes the immobile (c, d) and mobile (e, f) biomarkers and the degradation rates. The solid and dashed lines refer to the enzyme-based 1D flow model, the dotted to the Monod-based, while the long-dashed lines refer to the conservative transport of cis-DCE. In the plots, the concentration of 16S rRNA gene copies corresponds to the number of *Dehalococcoides* cells.

The 1D scenario-based RTMs simulating the transient (i.e., 10 years) and steady-state (i.e., 40 years) stages of the development of a cis-DCE plume in a homogeneous aquifer provided patterns of CEs and biomarkers that could be expected in real contaminated sites during sampling activities (Figure 3). These models included advective-dispersive transport, sorption of CEs and ethene, and partitioning of *Dehalococcoides* between the aqueous and solid phases.

The continuous release of cis-DCE from the source over 10 years created a plume extending to around 900 m. Both EBK and Monod models showed that the CE plume underwent significant sequential degradation (Figure 3a), causing the almost complete depletion of cis-DCE within the first 500 m and the accumulation of VC, ethene, and chloride. RD shaping the CE patterns relates to the rapid growth of immobile *Dehalococcoides* (Figure 3c), which reached the assumed maximum. Consequently, mobile *Dehalococcoides* released in groundwater reached a constant concentration (Figure 3e). As a result, the aquifer already attained its full dechlorination potential and consequently steady-state degradation rates in the first 600 m (Figure 3g,h), even when the plume was still elongated from the source. Immobile and mobile *tceA* transcripts and TceA enzymes were mainly upregulated where the cis-DCE conversion was predominant (Figure 3c,e). Even though both immobile and mobile *vcrA* transcripts and VcrA enzymes reached the highest levels where the conversion of VC to ethene prevailed (Figure 3c,e), they started to be upregulated during cis-DCE degradation. In the plume fringe (i.e., 600–900 m), immobile *Dehalococcoides* were still growing due to the downstream shift of the plume front. This transient growth condition caused an overproduction of mobile *Dehalococcoides*, resulting in a local upregulation of the transcripts and enzymes. These newly produced biomarkers were added to those already flowing in the aquifer. Mobile transcripts and enzymes were downregulated rapidly, while *Dehalococcoides* remained at a higher concentration. As already observed under microcosm conditions (Figure 2), this evidence suggests that the proposed enzyme-based kinetic equation framework was able to model the growth and subsequent inactivation of bacteria due to CE limitation, as previously observed in the literature.^76^ This condition is reached when CE concentrations are very low (i.e., in the order of a few μmol/L), as in the plume fringe, where the combination of hydrodynamic dispersion and degradation reduce contaminant levels.^77,78^ Eventually, the bacteria produced along the plume and transported downstream by groundwater flow die off without CEs, after becoming inactive immediately outside the fringe.

After 40 years, the simulated plume reached a steady state, representing the condition when biodegradation equilibrates the continuous release of cis-DCE from the source. The simulated CE patterns (Figure 3b) reflect those typically observed in CE plumes,^22,75,79,80^ where parent compounds sequentially degrade, producing daughter products downstream from the contaminant source. cis-DCE degradation occurred within the first 600 m, whereas VC turnover became the predominant process after 500 m (see the RD rates in Figure 3h). This sequence of dechlorination reactions led to the progressive accumulation of ethene and chloride along the flowline. At steady-state, the immobile *Dehalococcoides* (Figure 3d) remained consistently at the assumed maximum. Accordingly, mobile *Dehalococcoides* (Figure 3f) reached a homogeneous concentration along the whole plume. The immobile and mobile transcripts and enzymes of each functional gene were mainly upregulated, where the CE primarily activating its expression was predominant (i.e., cis-DCE for *tceA*, and VC for *vcrA*, respectively). Similarly to the 10-year scenario, *tceA* transcripts and TceA enzymes were not completely downregulated where cis-DCE was absent, and *vcrA* transcripts and VcrA enzymes started being upregulated during cis-DCE degradation. These modeled patterns can be expected in CE plumes if the considered *RDase* genes act as homologous functional genes.

### Relating Dechlorination Rates and Biomarkers

**Figure 4:**
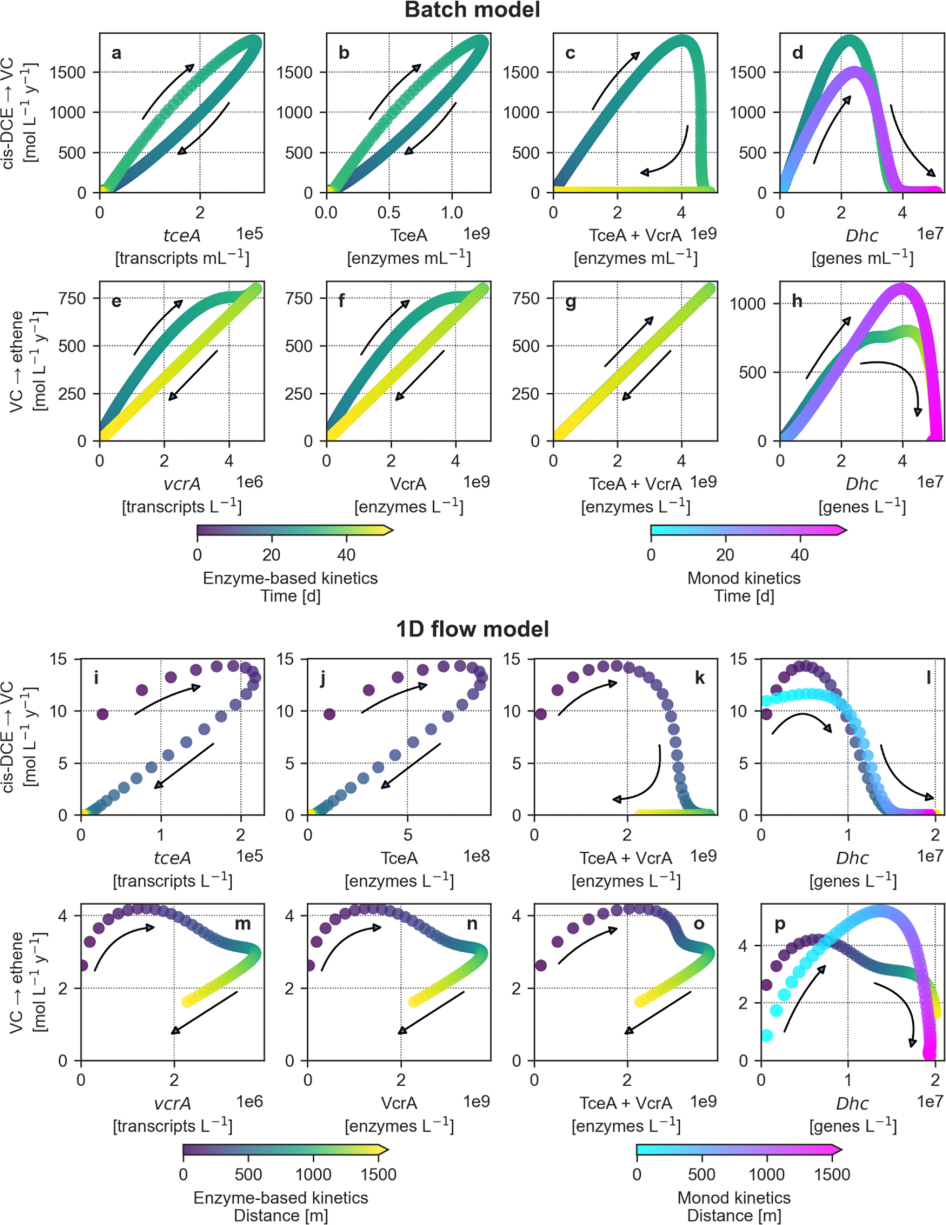
Relationships between biomarker levels and degradation rates of the two transformation steps of cis-DCE, via VC, to ethene, as shown for the batch model (a-h) and the 1D flow model at steady-state (i-p). Each plot describes how the biomarker-rate relationship varies over time or distance (color scales) for the batch model and 1D flow model, respectively. The linear correlation and degree of hysteresis (i.e., how different the ascending and descending parts of each curve are) are quantified by the Pearson coefficients (*r*) and the hysteresis loop areas (HLAs) in Tables S4 and S5, respectively.

Since biomarker levels have been considered reliable predictors for RD rates,^11,12,16,27,81,82^ we investigated these relationships and the consistency of this assumption by comparing the simulated transcripts, enzymes, and *Dehalococcoides* levels, and the RD rates under both microcosm and steady-state flow conditions (Figure 4). Note that groundwater samples collected during monitoring contain mobile biomarkers, whereas the overall degradation rates depend on the contribution of both mobile and immobile *Dehalococcoides* (Figure S2). Therefore, we analyzed the biomarker-rate relationships, considering mobile concentrations and overall RD rates.

Under microcosm conditions, transcripts (Figure 4a,e) were highly correlated (*r* = 0.986) with the degradation rates of the corresponding CE that predominantly regulated their transcription (i.e., cis-DCE for *tceA* transcripts and VC for *vcrA* transcripts). The same associations were observed between the rates and enzymes (Figure 4b,f). The high correlation between both biomarker levels and rates relates to the hypothesis that the metabolic regulation of the organohalide respiration in *Dehalococcoides* occurs at the transcriptional level.^62^ The biomarker-rate relations were slightly hysteretic (HLA = 0.164–0.167), meaning that upregulation and downregulation phases have different shapes (i.e., concave and convex or vice versa). This behavior suggests that the rates of each dechlorination step were partially linked to the expression of both *tceA* and *vcrA*, behaving as homologous genes. In addition to fitting the proposed enzyme-based model to real data, this hypothesis is further supported by experimental observations in the literature.^9,24,50−55^ For this reason, we also compared the sum of both enzymes to the overall rate (Figure 4c,g), and the resulting relationships appear to be significantly different. This relationship was markedly hysteretic (HLA = 0.587) in the case of cis-DCE degradation, whereas the total enzymes showed a robust positive linear correlation with the VC degradation rate (*r* = 0.999). This discrepancy likely relates to the combined contribution of both homologous enzymes to each degradation step. In the first case, the production of both TceA and VcrA enzymes continued due to the presence of VC, even after the complete disappearance of cis-DCE. In the second case, VC degradation occurred at low rates when cis-DCE was the predominant species activating *RDase* gene expression (Figure S1). Afterward, VC increased considerably and became the only CE regulating the enzymes responsible for its degradation, resulting in a linear correlation (Figure 4g). The *Dehalococcoides* modeled through both kinetic approaches (Figure 4d,h) were positively correlated with rates, until the latter reached their peak values. Afterward, bacteria and degradation rates decorrelate completely (Table S4). The growth of *Dehalococcoides* on both cis-DCE and VC explains these nonlinear relationships. Furthermore, their concentrations remained high even after the complete consumption of both CEs, in a state of inactivity.^76^ As a result, *Dehalococcoides* showed marked nonlinear relationships with rates; thus, these bacteria proved to be unreliable predictors.^81^ Nonlinear hysteretic relationships between biomarkers and rates similar to the results obtained in our batch model have been found in studies on modeling nitrate^56^ and pesticides^83^ biodegradation. Hysteretic relationships apparently depend on complex dynamics underlying the degradation processes such as intricate metabolic pathways (e.g., homologous gene expression), the asynchronism of substrate dynamics, biomarker production, or decay.

Under flow conditions, biomarker-rate relationships became even more hysteretic (Figure 4i–p; Table S5). Near the source, the upregulation of *tceA* transcripts and TceA enzymes due to the continuous release of cis-DCE increased the degradation rates of both cis-DCE and VC (Figure 4i,j). The cis-DCE dechlorination rate kept increasing until 100 m. Because of advective-dispersive transport, the mobile *tceA* transcripts and TceA enzymes released in groundwater kept increasing until about 200 m, locally causing decorrelation between transcript/enzyme levels and rates and consequently nonlinear hysteretic biomarker-rate relationships. Downstream, cis-DCE degradation downregulated *tceA* expression and decreased cis-DCE turnover rates to zero within 750 m (Figure 4i,j). The contemporary presence of both cis-DCE and VC induced the production and release of mobile *vcrA* transcripts and VcrA enzymes, which remained upregulated until about 1000 m. However, the downregulation of *tceA* expression induced a decrease of the VC dechlorination rates, also causing the local decorrelation visible in Figure 4m,n. From about 1000 m, the *vcrA* expression and the VC degradation rates slightly declined because of the decreasing VC concentration. We additionally compared the degradation rates and the combination of both enzymes (Figure 4k,o) to investigate whether the sum of homologous enzymes could be a reliable predictor of the RD rates. In both cases, the biomarker-rate relationships were markedly hysteretic (i.e., HLA is 0.722 for the cis-DCE degradation rate and 0.539 for the VC degradation rate): in the first case, because of the advective-dispersive transport of the mobile *tceA* transcripts and TceA enzymes and the contribution to *vcrA* expression yielded by the presence of VC; in the second case, due to the downregulation of the *tceA* expression induced by the disappearance of cis-DCE. RD rates were independent of the mobile *Dehalococcoides* levels (Table S4) in both the enzyme-based (Figure 4l) and the Monod models (Figure 4p) because of the growth as immobile biomass on both CEs and subsequent release and transport in the aquifer.

Similar nonlinear biomarker-rate relationships under flow conditions have been observed in the literature^84^ and attributed to the electron acceptor limitation. In our case, the behavior of *tceA* and *vcrA* as homologous genes and the effect of advective-dispersive transport on mobile biomarkers further complicated the biomarker-rate relationships. In fact, CEs degrade owing to the combined effect of mobile and immobile *Dehalococcoides*, which leads to more regular biomarker-rate relationships (Figure S3). Instead, groundwater samples contain only mobile *Dehalococcoides*. Consequently, the interpretation of observed biomarker patterns and the prediction of RD rates became even more challenging without models such as EBK.

### Implications for Biogeochemical Modeling and Outlook

The enzyme-based kinetics we implemented and calibrated to experimental microcosm data^58^ enabled us to simulate the *RDase* functional gene expression during the organohalide respiration in *Dehalococcoides*. We expanded the previous attempts^49,56,83^ by considering the *RDase* functional genes as homologous, which is a behavior previously observed in *Dehalococcoides.*(9,24,50−55) In addition to providing better fitting using the same parameter ranges and observation weights, considering homologous genes made our approach more flexible in modeling metabolic regulation dynamics in OHRB communities possibly harboring homologous *RDase* functional genes. The EBK model proved as effective as the Monod model in fitting the CEs, ethene, chloride, and bacteria time series; additionally, it reproduced the temporal patterns of *tceA* and *vcrA* transcripts and provided corresponding RDase enzyme levels in line with literature measurements.^16^

This novel EBK model was successfully integrated for the first time into a full-aquifer-scale 1D flow model simulating the development of a cis-DCE plume in an aquifer. This scenario-based RTM allowed simulating the expected spatial patterns of *RDase* transcripts and enzymes in addition to the CEs distributions within the plume. In the case of typically observed CE patterns in plumes,^22,76,79,80^*tceA* expression is expected to be upregulated where cis-DCE degradation is predominant, whereas the *vcrA* gene is mainly expressed where VC is dominant. In the case that these functional genes are homologous, they may not downregulate completely, even when the CE that mostly upregulates each of them is absent.

It was possible to relate RD rates with transcript and enzyme levels of *RDase* genes under both microcosm and flow conditions. As in previous attempts,^56,83,84^ these relationships were markedly nonlinear hysteretic. Moreover, the effect of advective-dispersive transport on mobile biomarkers further increased the level of hysteresis under the field conditions. These results challenge the widely accepted yet simplistic concept of linearity in biomarker-rate relationships^11,12,16,27,82^ and call for enzyme-based kinetics modeling to infer degradation rates from biomarkers measurements.

Our model considers *Dehalococcoides* as the growing biomass because the number of 16S rRNA gene copies represents the actual abundance of bacteria.^85^ In this way, mobile *Dehalococcoides* could be modeled as individual cells partially flowing within the saturated porous media. However, CE plumes often contain PCE and/or TCE as parent compounds, which can be degraded by more than one species. As a consequence, the original formulation may need to be adjusted to link transcripts, enzymes, and subsequently rates directly to *RDase* genes to include the preceding PCE-to-TCE and TCE-to-cis-DCE degradation steps.

The proposed EBK is in fact able to thoroughly mimic the complete metabolic regulation underlying the organohalide respiration in *Dehalococcoides* and to model the spatiotemporal patterns of the corresponding genes, transcripts, and enzymes. However, parameters in the quasi-steady-state translation equations were completely unconstrained because of the lack of accurate protein measurements, which are nowadays possible also for oligotrophic groundwaters.^86^ Thus, highly resolved time series of functional gene DNA, mRNA, and protein measurements are essential to improve model constraint, parametrization, and validation.

Microcosm experiments to study the complete degradation of PCE to ethene are needed to expand the enzyme-based kinetics. Direct measurements of *RDase* gene DNA, mRNA, and proteins in both microcosms and plumes would clarify whether post-transcriptional and/or post-translational regulation may be relevant for RDase enzyme production, as already observed in OHRB.^87,88^ This evidence would additionally help refine the enzyme-based kinetics, which now considers both transcription and translation in the steady state.

Overall, this study demonstrated that this novel and integrative modeling approach offers a more thorough mechanistic understanding of the observed spatiotemporal patterns of pollutants and biomarkers in groundwater systems. When constrained by chemical and biomolecular measurements, such RTMs incorporating enzyme-based kinetics provide a more reliable simulation of the biodegradation of CEs. As compound-specific isotope-based models have represented a valid aid for plume investigation and monitoring,^38−40^ this modeling approach will make RTMs better constrained and support the definition of more effective monitoring strategies of CE plumes, either during monitored natural attenuation or active bioremediation.

## Data Availability

The data, model results, PHREEQC input files, and Jupyter notebook for visualization related to this study are stored on the 4TU.ResearchData repository and can be accessed via https://data.4tu.nl/datasets/96405c3a-aad9-4904-b6ea-5aabe6280e15.

## Supporting Information Available

Supporting descriptions of microcosm experiments (Section S1); calibration method (Section S2); and approaches to modeling of retardation (Section S3); bacteria transport in porous media (Section S4); octanol–water partitioning coefficients (Table S1); calibrated kinetic parameters (Table S2); model features (Table S3); Pearson correlations (Table S4); hysteresis loop areas (Table S5) for biomarker-rate relations; nonhomologous functional gene- scenario batch model (Figure S1); and additional results of 1D scenario-based reactive transport model (Figures S2 and S3) (PDF)

## References

[ref1] Gerba C. P. Environmental Toxicology. In Environmental and Pollution Science; Brusseau M. L.; Pepper I. L.; Gerba C. P., Eds.; Academic Press, 2019; p 633.

[ref2] Leys D.; Adrian L.; Smidt H. Organohalide respiration: microbes breathing chlorinated molecules. Philos. Trans. R. Soc., B 2013, 368 (1616), 8–10. 10.1098/rstb.2012.0316. PMC3638457 23479746

[ref3] Vogel T. M.; Criddle C. S.; McCarty P. L. ES&T Critical Reviews: Transformations of halogenated aliphatic compounds. Environ. Sci. Technol. 1987, 21 (8), 722–736. 10.1021/es00162a001. 19995052

[ref4] Dolinová I.; Štrojsová M.; Černík M.; Němeček J.; Macháčková J.; Ševců A. Microbial degradation of chloroethenes: a review. Environ. Sci. Pollut. Res. 2017, 24 (15), 13262–13283. 10.1007/s11356-017-8867-y. 28378313

[ref5] Dolfing J. Energetic considerations in organohalide respiration. In Organohalide-Respiring Bacteria; Adrian L.; Löffler F. E., Eds.; Springer: Berlin, 2016; p 632.

[ref6] Rupakula A.; Kruse T.; Boeren S.; Holliger C.; Smidt H.; Maillard J. The restricted metabolism of the obligate organohalide respiring bacterium *Dehalobacter restrictus*: lessons from tiered functional genomics. Philos. Trans. R. Soc., B 2013, 368 (1616), 20120325 10.1098/rstb.2012.0325. PMC3638465 23479754

[ref7] Heavner G. L. W.; Mansfeldt C. B.; Debs G. E.; Hellerstedt S. T.; Rowe A. R.; Richardson R. E. Biomarkers’ Responses to Reductive Dechlorination Rates and Oxygen Stress in Bioaugmentation Culture KB-1. Microorganisms 2018, 6 (1), 1–13. 10.3390/microorganisms6010013. PMC5874627 29419787

[ref8] Nakamura R.; Obata T.; Nojima R.; Hashimoto Y.; Noguchi K.; Ogawa T.; Yohda M. Functional Expression and Characterization of Tetrachloroethene Dehalogenase from *Geobacter* sp. Front. Microbiol. 2018, 9 (8), 1–7. 10.3389/fmicb.2018.01774. 30147676 PMC6095959

[ref9] Yan J.; Wang J.; Villalobos Solis M. I.; Jin H.; Chourey K.; Li X.; Yang Y.; Yin Y.; Hettich R. L.; Löffler F. E. Respiratory Vinyl Chloride Reductive Dechlorination to Ethene in TceA-Expressing *Dehalococcoides mccartyi*. Environ. Sci. Technol. 2021, 55 (8), 4831–4841. 10.1021/acs.est.0c07354. 33683880

[ref10] Cimmino L.; Schmid A. W.; Holliger C.; Maillard J. Stoichiometry of the Gene Products From the Tetrachloroethene Reductive Dehalogenase Operon *pceABCT*. Front. Microbiol. 2022, 13 (2), 1–13. 10.3389/fmicb.2022.838026. PMC8905343 35283847

[ref11] Rahm B. G.; Richardson R. E. Correlation of Respiratory Gene Expression Levels and Pseudo-Steady State PCE Respiration Rates in *Dehalococcoides ethenogenes*. Environ. Sci. Technol. 2008, 42 (11), 416–421. 10.1021/es071455s. 18284140

[ref12] Rahm B. G.; Richardson R. E. *Dehalococcoides*’ Gene Transcripts As Quantitative Bioindicators of Tetrachloroethene, Trichloroethene, and *cis*-1,2-Dichloroethene Dehalorespiration Rates. Environ. Sci. Technol. 2008, 42 (14), 5099–5105. 10.1021/es702912t. 18754354

[ref13] Maillard J.; Charnay M. P.; Regeard C.; Rohrbach-Brandt E.; Rouzeau-Szynalski K.; Rossi P.; Holliger C. Reductive dechlorination of tetrachloroethene by a stepwise catalysis of different organohalide respiring bacteria and reductive dehalogenases. Biodegradation 2011, 22 (5), 949–960. 10.1007/s10532-011-9454-4. 21243405

[ref14] Rowe A. R.; Heavner G. L.; Mansfeldt C. B.; Werner J. J.; Richardson R. E. Relating Chloroethene Respiration Rates in *Dehalococcoides* to Protein and mRNA Biomarkers. Environ. Sci. Technol. 2012, 46 (17), 9388–9397. 10.1021/es300996c. 22812668

[ref15] Matturro B.; Majone M.; Aulenta F.; Rossetti S. Correlations between maximum reductive dechlorination rates and specific biomass parameters in *Dehalococcoides mccartyi* consortia enriched on chloroethenes PCE, TCE and cis-1,2-DCE. FEMS Microbiol. Ecol. 2021, 97 (6), 1–9. 10.1093/femsec/fiab064. 33899920

[ref16] Michalsen M. M.; Murdoch F. K.; Löffler F. E.; Wilson J.; Hatzinger P. B.; Istok J. D.; Mullins L.; Hill A.; Murdoch R. W.; Condee C.; Kucharzyk K. H. Quantitative Proteomics and Quantitative PCR as Predictors of *cis*-1,2-Dichlorethene and Vinyl Chloride Reductive Dechlorination Rates in Bioaugmented Aquifer Microcosms. ACS ES&T Eng. 2022, 2 (1), 43–53. 10.1021/acsestengg.1c00207.

[ref17] Schaefer C. E.; Condee C. W.; Vainberg S.; Steffan R. J. Bioaugmentation for chlorinated ethenes using *Dehalococcoides* sp.: Comparison between batch and column experiments. Chemosphere 2009, 75 (2), 141–148. 10.1016/j.chemosphere.2008.12.041. 19171368

[ref18] Lovley D. R. Cleaning up with genomics: applying molecular biology to bioremediation. Nat. Rev. Microbiol. 2003, 1 (1), 35–44. 10.1038/nrmicro731. 15040178

[ref19] Desai C.; Pathak H.; Madamwar D. Advances in molecular and “-omics” technologies to gauge microbial communities and bioremediation at xenobiotic/anthropogen contaminated sites. Bioresour. Technol. 2010, 101 (6), 1558–1569. 10.1016/j.biortech.2009.10.080. 19962886

[ref20] Meckenstock R. U.; Elsner M.; Griebler C.; Lueders T.; Stumpp C.; Aamand J.; Agathos S. N.; Albrechtsen H. J.; Bastiaens L.; Bjerg P. L.; Boon N.; Dejonghe W.; Huang W. E.; Schmidt S. I.; Smolders E.; Sørensen S. R.; Springael D.; Van Breukelen B. M. Biodegradation: Updating the Concepts of Control for Microbial Cleanup in Contaminated Aquifers. Environ. Sci. Technol. 2015, 49 (12), 7073–7081. 10.1021/acs.est.5b00715. 26000605

[ref21] Jugder B. E.; Ertan H.; Bohl S.; Lee M.; Marquis C. P.; Manefield M. Organohalide Respiring Bacteria and Reductive Dehalogenases: Key Tools in Organohalide Bioremediation. Front. Microbiol. 2016, 7 (3), 1–12. 10.3389/fmicb.2016.00249. 26973626 PMC4771760

[ref22] Hunkeler D.; Abe Y.; Broholm M. M.; Jeannottat S.; Westergaard C.; Jacobsen C. S.; Aravena R.; Bjerg P. L. Assessing chlorinated ethene degradation in a large scale contaminant plume by dual carbon–chlorine isotope analysis and quantitative PCR. J. Contam. Hydrol. 2011, 119 (1–4), 69–79. 10.1016/j.jconhyd.2010.09.009. 21030108

[ref23] Damgaard I.; Bjerg P. L.; Bælum J.; Scheutz C.; Hunkeler D.; Jacobsen C. S.; Tuxen N.; Broholm M. M. Identification of chlorinated solvents degradation zones in clay till by high resolution chemical, microbial and compound specific isotope analysis. J. Contam. Hydrol. 2013, 146, 37–50. 10.1016/j.jconhyd.2012.11.010. 23357226

[ref24] Hermon L.; Hellal J.; Denonfoux J.; Vuilleumier S.; Imfeld G.; Urien C.; Ferreira S.; Joulian C. Functional Genes and Bacterial Communities During Organohalide Respiration of Chloroethenes in Microcosms of Multi-Contaminated Groundwater. Front. Microbiol. 2019, 10 (2), 1–16. 10.3389/fmicb.2019.00089. 30809199 PMC6379275

[ref25] Ottosen C. B.; Rønde V.; McKnight U. S.; Annable M. D.; Broholm M. M.; Devlin J. F.; Bjerg P. L. Natural attenuation of a chlorinated ethene plume discharging to a stream: Integrated assessment of hydrogeological, chemical and microbial interactions. Water Res. 2020, 186, 1–14. 10.1016/j.watres.2020.116332. 32871289

[ref26] Damgaard I.; Bjerg P. L.; Jacobsen C. S.; Tsitonaki A.; Kerrn-Jespersen H.; Broholm M. M. Performance of Full-Scale Enhanced Reductive Dechlorination in Clay Till. Groundw. Monit. Remediat. 2013, 33 (1), 48–61. 10.1111/j.1745-6592.2012.01405.x.

[ref27] Liang Y.; Liu X.; Singletary M. A.; Wang K.; Mattes T. E. Relationships between the Abundance and Expression of Functional Genes from Vinyl Chloride (VC)-Degrading Bacteria and Geochemical Parameters at VC-Contaminated Sites. Environ. Sci. Technol. 2017, 51 (21), 12164–12174. 10.1021/acs.est.7b03521. 28981261

[ref28] Heavner G. L. W.; Mansfeldt C. B.; Wilkins M. J.; Nicora C. D.; Debs G. E.; Edwards E. A.; Richardson R. E. Detection of Organohalide-Respiring Enzyme Biomarkers at a Bioaugmented TCE-Contaminated Field Site. Front. Microbiol. 2019, 10 (6), 1–12. 10.3389/fmicb.2019.01433. 31316484 PMC6610324

[ref29] Murray A.; Maillard J.; Rolle M.; Broholm M.; Holliger C. Impact of iron- and/or sulfate-reduction on a *cis*-1,2-dichloroethene and vinyl chloride respiring bacterial consortium: experiments and model-based interpretation. Environ. Sci.: Processes Impacts 2020, 22 (3), 740–750. 10.1039/C9EM00544G. 32003373

[ref30] Clement T. P.; Johnson C. D.; Sun Y.; Klecka G. M.; Bartlett C. Natural attenuation of chlorinated ethene compounds: model development and field-scale application at the Dover site. J. Contam. Hydrol. 2000, 42 (2–4), 113–140. 10.1016/S0169-7722(99)00098-4.

[ref31] Ling M.; Rifai H. S. Modeling Natural Attenuation with Source Control at a Chlorinated Solvents Dry Cleaner Site. Groundwater Monit. Rem. 2007, 27 (1), 108–121. 10.1111/j.1745-6592.2006.00129.x.

[ref32] Antelmi M.; Mazzon P.; Höhener P.; Marchesi M.; Alberti L. Evaluation of MNA in a Chlorinated Solvents-Contaminated Aquifer Using Reactive Transport Modeling Coupled with Isotopic Fractionation Analysis. Water 2021, 13 (21), 1–22. 10.3390/w13212945.

[ref33] Wood R. C.; Huang J.; Goltz M. N. Modeling Chlorinated Solvent Bioremediation Using Hydrogen Release Compound (HRC). Biorem. J. 2006, 10 (3), 129–141. 10.1080/10889860600911947.

[ref34] Manoli G.; Chambon J. C.; Bjerg P. L.; Scheutz C.; Binning P. J.; Broholm M. M. A remediation performance model for enhanced metabolic reductive dechlorination of chloroethenes in fractured clay till. J. Contam. Hydrol. 2012, 131 (1–4), 64–78. 10.1016/j.jconhyd.2012.01.004. 22343011

[ref35] Viotti P.; Di Palma P. R.; Aulenta F.; Luciano A.; Mancini G.; Papini M. P. Use of a reactive transport model to describe reductive dechlorination (RD) as a remediation design tool: application at a CAH-contaminated site. Environ. Sci. Pollut. Res. 2014, 21 (2), 1514–1527. 10.1007/s11356-013-2035-9. 23933954

[ref36] Sprocati R.; Flyvbjerg J.; Tuxen N.; Rolle M. Process-based modeling of electrokinetic-enhanced bioremediation of chlorinated ethenes. J. Hazard. Mater. 2020, 397 (1), 1–14. 10.1016/j.jhazmat.2020.122787. 32388097

[ref37] Sprocati R.; Rolle M. Integrating Process-Based Reactive Transport Modeling and Machine Learning for Electrokinetic Remediation of Contaminated Groundwater. Water Resour. Res. 2021, 57 (8), 1–22. 10.1029/2021WR029959.

[ref38] van Breukelen B. M.; Hunkeler D.; Volkering F. Quantification of Sequential Chlorinated Ethene Degradation by Use of a Reactive Transport Model Incorporating Isotope Fractionation. Environ. Sci. Technol. 2005, 39 (11), 4189–4197. 10.1021/es048973c. 15984799

[ref39] van Breukelen B. M.; Thouement H. A. A.; Stack P. E.; Vanderford M.; Philp P.; Kuder T. Modeling 3D-CSIA data: Carbon, chlorine, and hydrogen isotope fractionation during reductive dechlorination of TCE to ethene. J. Contam. Hydrol. 2017, 204 (7), 79–89. 10.1016/j.jconhyd.2017.07.003. 28764859

[ref40] Höhener P. Simulating stable carbon and chlorine isotope ratios in dissolved chlorinated groundwater pollutants with BIOCHLOR-ISO. J. Contam. Hydrol. 2016, 195, 52–61. 10.1016/j.jconhyd.2016.11.002. 27894785

[ref41] Chambon J. C.; Bjerg P. L.; Scheutz C.; Baelum J.; Jakobsen R.; Binning P. J. Review of reactive kinetic models describing reductive dechlorination of chlorinated ethenes in soil and groundwater. Biotechnol. Bioeng. 2013, 110 (1), 1–23. 10.1002/bit.24714. 22926627

[ref42] Yu S.; Dolan M. E.; Semprini L. Kinetics and Inhibition of Reductive Dechlorination of Chlorinated Ethylenes by Two Different Mixed Cultures. Environ. Sci. Technol. 2005, 39 (1), 195–205. 10.1021/es0496773. 15667095

[ref43] Haest P. J.; Springael D.; Smolders E. Modelling reactive CAH transport using batch experiment degradation kinetics. Water Res. 2010, 44 (9), 2981–2989. 10.1016/j.watres.2010.02.031. 20303564

[ref44] Malaguerra F.; Chambon J. C.; Bjerg P. L.; Scheutz C.; Binning P. J. Development and Sensitivity Analysis of a Fully Kinetic Model of Sequential Reductive Dechlorination in Groundwater. Environ. Sci. Technol. 2011, 45 (19), 8395–8402. 10.1021/es201270z. 21877704

[ref45] Chen M.; Abriola L. M.; Amos B. K.; Suchomel E. J.; Pennell K. D.; Löffler F. E.; Christ J. A. Microbially enhanced dissolution and reductive dechlorination of PCE by a mixed culture: Model validation and sensitivity analysis. J. Contam. Hydrol. 2013, 151, 117–130. 10.1016/j.jconhyd.2013.05.005. 23774611

[ref46] Bælum J.; Scheutz C.; Chambon J. C.; Jensen C. M.; Brochmann R. P.; Dennis P.; Laier T.; Broholm M. M.; Bjerg P. L.; Binning P. J.; Jacobsen C. S. The impact of bioaugmentation on dechlorination kinetics and on microbial dechlorinating communities in subsurface clay till. Environ. Pollut. 2014, 186, 149–157. 10.1016/j.envpol.2013.11.013. 24374375

[ref47] Schneidewind U.; Haest P. J.; Atashgahi S.; Maphosa F.; Hamonts K.; Maesen M.; Calderer M.; Seuntjens P.; Smidt H.; Springael D.; Dejonghe W. Kinetics of dechlorination by *Dehalococcoides mccartyi* using different carbon sources. J. Contam. Hydrol. 2014, 157, 25–36. 10.1016/j.jconhyd.2013.10.006. 24275111

[ref48] Heavner G. L. W.; Rowe A. R.; Mansfeldt C. B.; Pan J. K.; Gossett J. M.; Richardson R. E. Molecular Biomarker-Based Biokinetic Modeling of a PCE-Dechlorinating and Methanogenic Mixed Culture. Environ. Sci. Technol. 2013, 47 (8), 3724–3733. 10.1021/es303517s. 23363057

[ref49] Bælum J.; Chambon J. C.; Scheutz C.; Binning P. J.; Laier T.; Bjerg P. L.; Jacobsen C. S. A conceptual model linking functional gene expression and reductive dechlorination rates of chlorinated ethenes in clay rich groundwater sediment. Water Res. 2013, 47 (7), 2467–2478. 10.1016/j.watres.2013.02.016. 23490098

[ref50] Futagami T.; Goto M.; Furukawa K. Biochemical and genetic bases of dehalorespiration. Chem. Rec. 2008, 8 (1), 1–12. 10.1002/tcr.20134. 18302277

[ref51] Hug L. A. Diversity, evolution, and environmental distribution of reductive dehalogenase genes. In Organohalide-Respiring Bacteria; Adrian L.; Löffler F. E., Eds.; Springer: Berlin, 2016; p 632.

[ref52] Zhao S.; Ding C.; He J. Genomic characterization of *Dehalococcoides mccartyi* strain 11a5 reveals a circular extrachromosomal genetic element and a new tetrachloroethene reductive dehalogenase gene. FEMS Microbiol. Ecol. 2017, 93 (4), 1–11. 10.1093/femsec/fiw235. 27856620

[ref53] Saiyari D. M.; Chuang H. P.; Senoro D. B.; Lin T. F.; Whang L. M.; Chiu Y. T.; Chen Y. H. A review in the current developments of genus *Dehalococcoides*, its consortia and kinetics for bioremediation options of contaminated groundwater. Sustainable Environ. Res. 2018, 28 (4), 149–157. 10.1016/j.serj.2018.01.006.

[ref54] Yu Y.; Zhang Y.; Liu Y.; Lv M.; Wang Z.; Wen L. lian.; Li A. *In situ* reductive dehalogenation of groundwater driven by innovative organic carbon source materials: Insights into the organohalide-respiratory electron transport chain. J. Hazard. Mater. 2023, 452 (2), 1–15. 10.1016/j.jhazmat.2023.131243. 36989787

[ref55] Waller A. S.; Krajmalnik-Brown R.; Löffler F. E.; Edwards E. A. Multiple Reductive-Dehalogenase-Homologous Genes Are Simultaneously Transcribed during Dechlorination by *Dehalococcoides*-Containing Cultures. Appl. Environ. Microbiol. 2005, 71 (12), 8257–8264. 10.1128/AEM.71.12.8257-8264.2005. 16332811 PMC1317432

[ref56] Störiko A.; Pagel H.; Mellage A.; Cirpka O. A. Does It Pay Off to Explicitly Link Functional Gene Expression to Denitrification Rates in Reaction Models?. Front. Microbiol. 2021, 12 (6), 1–13. 10.3389/fmicb.2021.684146. PMC8250433 34220770

[ref57] Maillard J.; Willemin M. S. Regulation of organohalide respiration. In Advances in Microbial Physiology; Poole R. K., Ed.; Elsevier, 2019; p 514 10.1016/bs.ampbs.2019.02.002. 31126531

[ref58] Kranzioch I.; Ganz S.; Tiehm A. Chloroethene degradation and expression of *Dehalococcoides* dehalogenase genes in cultures originating from Yangtze sediments. Environ. Sci. Pollut. Res. 2015, 22 (4), 3138–3148. 10.1007/s11356-014-3574-4. 25233916

[ref59] Krasper L.; Lilie H.; Kublik A.; Adrian L.; Golbik R.; Lechner U. The MarR-Type Regulator Rdh2R Regulates *rdh* Gene Transcription in *Dehalococcoides mccartyi* Strain CBDB1. J. Bacteriol. 2016, 198 (23), 3130–3141. 10.1128/JB.00419-16. 27621279 PMC5105894

[ref60] He J.; Sung Y.; Krajmalnik-Brown R.; Ritalahti K. M.; Löffler F. E. Isolation and characterization of *Dehalococcoides* sp. strain FL2, a trichloroethene (TCE)- and 1,2-dichloroethene-respiring anaerobe. Environ. Microbiol. 2005, 7 (9), 1442–1450. 10.1111/j.1462-2920.2005.00830.x. 16104866

[ref61] Ingalls B. P. Mathematical Modeling in Systems Bology: An Introduction; MIT Press 2013.

[ref62] West K. A.; Lee P. K. H.; Johnson D. R.; Zinder S. H.; Alvarez-Cohen L. Global gene expression of *Dehalococcoides* within a robust dynamic TCE-dechlorinating community under conditions of periodic substrate supply. Biotechnol. Bioeng. 2013, 110 (5), 1333–1341. 10.1002/bit.24819. 23280440

[ref63] Mundle S. O. C.; Johnson T.; Lacrampe-Couloume G.; Pérez-De-Mora A.; Duhamel M.; Edwards E. A.; McMaster M. L.; Cox E.; Révész K.; Sherwood Lollar B. Monitoring Biodegradation of Ethene and Bioremediation of Chlorinated Ethenes at a Contaminated Site Using Compound-Specific Isotope Analysis (CSIA). Environ. Sci. Technol. 2012, 46 (3), 1731–1738. 10.1021/es202792x. 22201221

[ref64] Balkwill D. L.; Leach F. R.; Wilson J. T.; McNabb J. F.; White D. C. Equivalence of microbial biomass measures based on membrane lipid and cell wall components, adenosine triphosphate, and direct counts in subsurface aquifer sediments. Microb. Ecol. 1988, 16 (1), 73–84. 10.1007/BF02097406. 24201534

[ref65] Parkhurst D. L.; Appelo C. A. J. Description of Input and Examples for PHREEQC Version 3—A Computer Program for Speciation, Batch-Reaction, One-Dimensional Transport, and Inverse Geochemical Calculations; U.S. Geological Survey, 2013.

[ref66] Doherty J. PEST: Model-Independent Parameter Estimation—User Manual: 5th ed.; Watermark Numerical Computing 2004.

[ref67] Antoniou E. A.; Stuyfzand P. J.; van Breukelen B. M. Reactive transport modeling of an aquifer storage and recovery (ASR) pilot to assess long-term water quality improvements and potential solutions. Appl. Geochem. 2013, 35, 173–186. 10.1016/j.apgeochem.2013.04.009.

[ref68] Kruisdijk E.; van Breukelen B. M. Reactive transport modelling of push-pull tests: A versatile approach to quantify aquifer reactivity. Appl. Geochem. 2021, 131 (5), 1–16. 10.1016/j.apgeochem.2021.104998.

[ref69] Zech A.; Attinger S.; Bellin A.; Cvetkovic V.; Dagan G.; Dietrich P.; Fiori A.; Teutsch G. Evidence Based Estimation of Macrodispersivity for Groundwater Transport Applications. Groundwater 2023, 61 (3), 346–362. 10.1111/gwat.13252. 36114728

[ref70] Cápiro N. L.; Wang Y.; Hatt J. K.; Lebrón C. A.; Pennell K. D.; Löffler F. E. Distribution of Organohalide-Respiring Bacteria between Solid and Aqueous Phases. Environ. Sci. Technol. 2014, 48 (18), 10878–10887. 10.1021/es501320h. 25105899

[ref71] Hnatko J. P.; Yang L.; Pennell K. D.; Abriola L. M.; Cápiro N. L. Bioenhanced back diffusion and population dynamics of *Dehalococcoides mccartyi* strains in heterogeneous porous media. Chemosphere 2020, 254, 1–12. 10.1016/j.chemosphere.2020.126842. 32957273

[ref72] Carrera J.; Saaltink M. W.; Soler-Sagarra J.; Jingjing W.; Valhondo C. Reactive Transport: A Review of Basic Concepts with Emphasis on Biochemical Processes. Energies 2022, 15 (3), 1–47. 10.3390/en15030925.

[ref73] Takeuchi M.; Kawabe Y.; Watanabe E.; Oiwa T.; Takahashi M.; Nanba K.; Kamagata Y.; Hanada S.; Ohko Y.; Komai T. Comparative study of microbial dechlorination of chlorinated ethenes in an aquifer and a clayey aquitard. J. Contam. Hydrol. 2011, 124 (1–4), 14–24. 10.1016/j.jconhyd.2011.01.003. 21330000

[ref74] Elsgaard L. Reductive transformation and inhibitory effect of ethylene under methanogenic conditions in peat-soil. Soil Biol. Biochem. 2013, 60, 19–22. 10.1016/j.soilbio.2013.01.010.

[ref75] Maier T.; Schmidt A.; Güell M.; Kühner S.; Gavin A. C.; Aebersold R.; Serrano L. Q. Quantification of mRNA and protein and integration with protein turnover in a bacterium. Mol. Syst. Biol. 2011, 7 (1), 1–12. 10.1038/msb.2011.38. PMC3159969 21772259

[ref76] Mayer-Blackwell K.; Azizian M. F.; Green J. K.; Spormann A. M.; Semprini L. Survival of Vinyl Chloride Respiring *Dehalococcoides mccartyi* under Long-Term Electron Donor Limitation. Environ. Sci. Technol. 2017, 51 (3), 1635–1642. 10.1021/acs.est.6b05050. 28002948

[ref77] van Breukelen B. M.; Rolle M. Transverse Hydrodynamic Dispersion Effects on Isotope Signals in Groundwater Chlorinated Solvents’ Plumes. Environ. Sci. Technol. 2012, 46 (14), 7700–7708. 10.1021/es301058z. 22681629

[ref78] Wienkenjohann H.; Jin B.; Rolle M. Diffusive-Dispersive Isotope Fractionation of Chlorinated Ethenes in Groundwater: The Key Role of Incomplete Mixing and Its Multi-Scale Effects. Water Resour. Res. 2023, 59 (4), 1–18. 10.1029/2022WR034041.

[ref79] Atteia O.; Guillot C. Factors controlling BTEX and chlorinated solvents plume length under natural attenuation conditions. J. Contam. Hydrol. 2007, 90 (1–2), 81–104. 10.1016/j.jconhyd.2006.09.012. 17081653

[ref80] Atteia O.; Höhener P. Fast semi-analytical approach to approximate plumes of dissolved redox-reactive pollutants in heterogeneous aquifers. 2: Chlorinated ethenes. Adv. Water Resour. 2012, 46, 74–83. 10.1016/j.advwatres.2012.02.006.

[ref81] Lu X.; Wilson J. T.; Kampbell D. H. Relationship between *Dehalococcoides* DNA in ground water and rates of reductive dechlorination at field scale. Water Res. 2006, 40 (16), 3131–3140. 10.1016/j.watres.2006.05.030. 16889813

[ref82] Da Silva M. L.; Alvarez P. J. Exploring the Correlation between Halorespirer Biomarker Concentrations and TCE Dechlorination Rates. J. Environ. Eng. 2008, 134 (11), 895–901. 10.1061/(ASCE)0733-9372(2008)134:11(895).

[ref83] Rodriguez L. C.; Ingalls B.; Schwarz E.; Streck T.; Uksa M.; Pagel H. Gene-Centric Model Approaches for Accurate Prediction of Pesticide Biodegradation in Soils. Environ. Sci. Technol. 2020, 54 (21), 13638–13650. 10.1021/acs.est.0c03315. 33064475

[ref84] Störiko A.; Pagel H.; Mellage A.; Van Cappellen P.; Cirpka O. A. Denitrification-Driven Transcription and Enzyme Production at the River-Groundwater Interface: Insights From Reactive-Transport Modeling. Water Resour. Res. 2022, 58 (8), 1–23. 10.1029/2021WR031584.

[ref85] Ritalahti K. M.; Amos B. K.; Sung Y.; Wu Q.; Koenigsberg S. S.; Löffler F. E. Quantitative PCR Targeting 16S rRNA and Reductive Dehalogenase Genes Simultaneously Monitors Multiple *Dehalococcoides* Strains. Appl. Environ. Microbiol. 2006, 72 (4), 2765–2774. 10.1128/AEM.72.4.2765-2774.2006. 16597981 PMC1449079

[ref86] Corbera-Rubio F.; Laureni M.; Koudijs N.; Müller S.; van Alen T.; Schoonenberg F.; Lücker S.; Pabst M.; van Loosdrecht M. C. M.; van Halem D. Meta-omics profiling of full-scale groundwater rapid sand filters explains stratification of iron, ammonium and manganese removals. Water Res. 2023, 233 (11), 1–12. 10.1016/j.watres.2023.119805. 36868119

[ref87] Rowe A. R.; Mansfeldt C. B.; Heavner G. L.; Richardson R. E. R. Relating mRNA and protein biomarker levels in a *Dehalococcoides* and *Methanospirillum*-containing community. Appl. Microbiol. Biotechnol. 2015, 99 (5), 2313–2327. 10.1007/s00253-014-6220-7. 25467924

[ref88] Kruse T.; Smidt H.; Lechner U. Comparative genomics and transcriptomics of organohalide-respiring bacteria and regulation of *rdh* gene transcription. In Organohalide-Respiring Bacteria; Adrian L.; Löffler F. E., Eds.; Springer: Berlin, 2016; p 632.

