# Supplementary material for: Integrating Enzyme-Based Kinetics in Reactive Transport Models to Simulate Spatiotemporal Dynamics of Biomarkers during Chlorinated Ethene Degradation
Source: Environ Sci Technol. 2024 Nov 7;58(46):20642–53. doi: 10.1021/acs.est.4c07445 (PMC11580173; doi:10.1021/acs.est.4c07445)
Supplement: Supplementary file 1 — es4c07445_si_001.pdf [file es4c07445_si_001.pdf]

Supporting Information for

Integrating enzyme-based kinetics in reactive transport models to simulate spatiotemporal dynamics of biomarkers during chlorinated ethene degradation

Diego Di Curzio[1*], Michele Laureni[1], Mette Martina Broholm[2], David Weissbrodt[3], Boris M. van Breukelen[1]

[1] Delft University of Technology, Department of Water Management, Stevinweg 1, 2628 CN Delft, Netherlands.

[2] Technical University of Denmark, Department of Environmental and Resource Engineering, Bygningstorvet 115, 2800 Kongens Lyngby, Denmark.

[3] Norwegian University of Science and Technology, Department of Biotechnology and Food Science, Sem Sælandsvei 8, 7034 Trondheim, Norway.

* Corresponding author: D.DiCurzio@tudelft.nl

Summary:

Number of pages: **17**

Number of figures: **3**

Number of tables: **5**

Table of contents

List of sections

S1. Details regarding the microcosm experiments and data measurements..S3

S2. Kinetic model calibration S4

S3. Retardation of organic contaminants S5

S4. Modeling bacterial transport in porous media..S6

References S15

List of tables

Table S1. $logKow$ values from PubChem database of the US National Library of Medicine (https://pubchem.ncbi.nlm.nih.gov) used in the scenario-based RTMs..S6

Table S2. List of calibrated kinetic parameter values used in this study, with the corresponding unit and the explicit reference to the type of model..S8

Table S3. Relevant batch and 1D model features, such as initial and source concentration, and conversion factors..S9

Table S4. Pearson correlation coefficients (r) of the relationships between the biomarker levels and degradation rates of the two transformation steps of cis-DCE, via VC, to ethene, referred to panels in Fig. 4 in the main text..S13

Table S5. Hysteresis Loop Areas (HLAs) of the relationships between the biomarker levels and degradation rates of the two transformation steps of cis-DCE, via VC, to ethene, referred to panels in Fig. 4 in the main text. HLAs relate to the surface enclosed in hysteretic non-linear curves to measure the degree of hysteresis using the trapezoidal integration rule (Onoue, 2019). To remove the possible effect of the variability ranges of biomarker levels and degradation rates, each variable was previously scaled to the [0,1] range. In this way, the maximum reference area becomes equal to 1. ....S14

List of figures

Figure S1. Temporal patterns (solid lines) of chemical concentrations (a), biomarker levels (b), degradation rates (c), and transcription factor (d) in the microcosm experiment in Kranzioch et al. (2015), obtained through the enzyme-based kinetics and considering both tceA and vcrA as non-homologous. In the plots, the concentration of 16S rRNA gene copies corresponds to the number of Dehalococcoides cells. The black and grey dashed lines represent the quantification limits of Dehalococcoides 16S rRNA gene and the tceA and vcrA transcripts, respectively. Refer to the original paper for the error bars related to the standard deviation of all the chemical and biomarker measurements. S10

Figure S2. 1D scenario-based reactive transport model simulating two relevant stages of the evolution of a cis-DCE plume in groundwater: the elongation phase at 10 years, when the plume is in a transient state, and the steady-state condition at 40 years. In the plot, the sorbed chemicals (a,b), the degradation rates due to the immobile (c,d) and mobile (e,f) biomarkers, and the transcription factors (g,h). The solid lines refer to the enzyme-based 1D flow model, the dotted to the Monod-based, while the long-dashed lines ones to the conservative transport of cis-DCE. In the plots, the concentration of 16S rRNA gene copies corresponds to the number of Dehalococcoides cells..S11

Figure S3. Relationships between biomarker levels and degradation rates of the two transformation steps of cis-DCE, via VC, to ethene, related to the 1D flow model, considering only immobile biomarkers (a-h) and the combination of immobile and mobile biomarkers (i-p). Each plot describes how the biomarker-rate relationship distance (color scales)..S12

S1. Details regarding the microcosm experiments and data measurements

In Kranzioch et al. (2015), the authors performed a series of microcosm experiments to evaluate the dechlorination capability of sediments from the Yangtze River within the city of Chongqing in China and investigate dehalogenase gene expression.

In a preliminary experimental phase, 2-L glass bottles containing water, and 20 g of natural soils were enriched with auxiliary substrates (i.e., yeast extract, pyruvate, and acetate), as described in Kranzioch et al. (2013); then, one of the previously enriched cultures (i.e., YCQ4) was selected to investigate the temporal evolution of functional gene expression during the reductive dechlorination (RD) of chlorinated ethenes (CEs).

Two groups of experiments (i.e., 3 parallel test bottles each, one of which was a sterile control containing sodium azide) were conducted on YCQ4 culture: in the first, the culture was spiked with 50 μmol/L of PCE, while in the second with 50 μmol/L of cis-DCE. In all the cases, the bottles were incubated in the dark, at a temperature in the range of 22-24 °C.

During the experiments, aqueous phase samples were collected by stainless steel needles and analyzed with different methods to measure all the variables of interest: i) gas chromatography for chlorinated ethenes and ethene; ii) ion chromatography for chloride; iii) quantitative polymerases chain reaction (qPCR) targeting the 16S rRNA gene of *Dehalococcoides* spp.; iv) qPCR targeting specific functional genes for RDase genes, such as tetrachloroethene (*pceA*), trichloethene reductase (*tceA*), vinyl chloride reductase (*vcrA*, and *bvcA*) reductive dehalogenases; v) and quantitative reverse transcription PCR (RT-qPCR) for the corresponding mRNA transcripts. To overcome the limitations given by the short mRNA half-lives, the authors used a protocol to quantify the loss of nucleic acids based on an mRNA luciferase positive control. For further details about the analytical procedures, we refer to the original paper (Kranzioch et al., 2015).

The rapid degradation within 7 days of PCE to cis-DCE via TCE in the PCE-spiked microcosms was attributed to *Desulfitobacterium* spp., which were not measured. Instead,

the complete transformation of cis-DCE, via VC, to ethene occurring within 45 days was predominantly linked to the *Dehalococcoides* spp. (just *Dehalococcoides*, later on) respiration in both PCE- and cis-DCE-spiked cultures. The lack of measurements able to describe the microbial community dynamics behind the PCE degradation to cis-DCE via TCE prevented the use of data from the PCE-spiked culture because it would have meant implementing a partially unconstraint model. Therefore, we opted for using the data (i.e., 16S rRNA gene of *Dehalococcoides*, and functional gene transcripts) from the cis-DCE-spiked culture, which ensured the required complexity to implement the novel enzyme-based kinetics we propose.

This dataset is ideal to implement kinetic equations to model the link between RD and the underlying metabolic regulation dynamics in natural environments, such as contaminated aquifers: 1) natural sediments and bacterial community; 2) time series linking bacterial growth (i.e., 16S rRNA gene) and functional gene expression (i.e., at least one between mRNA and proteins). Furthermore, the selected data allowed focusing on the complete dechlorination of cis-DCE to ethene, via VC, which is the most interesting yet challenging to investigate set of dechlorination reactions.

It is worth pointing out that neither *pceA* nor *bvcA* mRNA was detected in the selected microcosm experiment; thus, their measurements (always below the detection limit) were neither considered in the modeling nor included in the plots in the main text.

S2. Kinetic model calibration

Both the enzyme-based and the Monod kinetics were calibrated using the Gauss-Marquardt-Levenberg method (Doherty, 2004). The weights (w_i) to be assigned to observations (C_i) during the model calibration were defined by the following equation (Hill, 1998), considering 5% accuracy:

$$w_i = \frac{1.96}{0.05 - C_i} \qquad \text{(S1)}$$

The target variables used for model calibration were chlorinated ethenes and chloride concentrations, the 16S rRNA gene of *Dehalococcoides*, and mRNA levels of *tceA* and *vcrA* genes for the enzyme-based kinetics. In contrast, only the chlorinated ethenes and bacteria concentrations were used for the Monod kinetics.

S3. Retardation of organic contaminants

In the scenario-based reactive transport models (RTMs), cis-DCE, VC, and ethene are considered to undergo retardation while transported in organic-carbon-containing saturated porous media, due to their hydrophobicity. The equation describing the retarded conservative one-dimensional advective-dispersive transport of the considered contaminants (Appelo and Postma, 2004) is the following:

$$\frac{\partial C_i}{\partial t} = \frac{D}{R_i}\frac{\partial^2 C_i}{\partial x^2} - \frac{v_w}{R_i}\frac{\partial C_i}{\partial t} \qquad \text{(S2)}$$

where C_i [mol L^{-1}] is the concentration of the retarded compound, D [m^2 s^{-1}] is the hydrodynamic dispersion (i.e., combination of mechanical dispersion and diffusion) coefficient, v_w [m s^{-1}] is the groundwater flow rate in the porous medium, while R_i [-] is the retardation factor.

The retardation factor affects both the hydrodynamic dispersion and the velocity of the solutes and is equal to:

$$R_i = 1 + K_d^i \qquad \text{(S3)}$$

where K_d^i [-] is the solid-water partitioning coefficient, which for the organic compounds is defined as follows, when the fraction of organic carbon the considered porous media is larger than 0.1% (i.e., $f_{oc} > 0.001$):

$$K_d^i = K_{oc}^i f_{oc} \frac{\rho_b}{\varepsilon_w} \qquad \text{(S4)}$$

where K_{oc}^i [L kg^{-1}] is the organic carbon-water partitioning coefficient, ρ_b [kg L^{-1}] is the bulk density of the saturated porous medium, and ε_w [-] is the water-filled porosity.

K^{i}_{oc} is highly correlated with the octanol-water partitioning coefficient (K^{i}_{ow}), which is defined experimentally, according to the relation proposed by Karickhoff (1981):

$$\log K^{i}_{oc} = \log K^{i}_{ow} - 0.35 \qquad \text{(S5)}$$

Tab. S1 shows the experimental $\log K_{ow}$values used in Eq. (S4) to infer the K_{oc} values for the scenario-based RTMs.

Table S1. $log\,K_{ow}$ values from PubChem database of the US National Library of Medicine (https://pubchem.ncbi.nlm.nih.gov) used in the scenario-based RTMs.

Organic chemical	$\log K_{ow}$	Reference
cis-DCE	1.86	Ran et al., 2002
VC	1.46	Sakuratani et al., 2007
ethene	1.13	Hansch et al., 1995

S4. Modeling bacterial transport in porous media

It has been observed experimentally that *Dehalococcoides* are predominantly associated with the solid phase during RD, with the fraction of mobile cells in the range 1.3-27% (Cápiro et al., 2014; Hnatko et al., 2020). In our scenario-based RTMs, this bacterial cell distribution between solid and aqueous phases was simulated through an approach similar to the one used in previous modeling endeavors (Clement et al., 1997; Phanikumar et al., 2005; Schaefer et al.; 2009), where *Dehalococcoides* are mainly immobile (B_{imm}), and growth on the solid matrix occurs either as biofilm or attached cells onto the pore surfaces, according to the following equation:

$$\frac{\rho_b}{\varepsilon_w}\frac{\partial B_{imm}}{\partial t} = f\left(\sum_i Y_i r^{i}_{RD}\right) - k_{dec} B_{imm} \qquad \text{(S6)}$$

where f [-] is the fraction of immobile *Dehalococcoides*, Y_i [mol of biomass mol of substrate^{-1}] is the yield factor, r^{i}_{RD} [mol L^{-1} s^{-1}] the dechlorination rate for each CE, and k_{dec} [s^{-1}] is the first-order coefficient for bacterial decay. Part of the *Dehalococcoides* cells growing on the solid matrix ($(1-f)$ [-]) is released in the pore water and undergoes advective-dispersive transport as well as decay:

$$\frac{\partial B_{mob}}{\partial t} = D\frac{\partial^2 B_{mob}}{\partial x^2} - v_w\frac{\partial B_{mob}}{\partial t} + (1-f)\left(\sum_i Y_i r_{RD}^i\right) - k_{dec}B_{mob} \tag{S7}$$

RD occurs due to the combined contribution of the immobile biomass actively growing on CEs and the mobile *Dehalococcoides* being released and passively flowing through the saturated pores:

$$r_{RD}^i = -\left(\mu_{max}^i\frac{B_{imm}}{\sum_i Y_i} + k_{max}^i B_{mob}\right)\frac{C_i}{C_i+K_i} \tag{S8}$$

where μ_{max}^i [s^{-1}] is the specific bacterial growth rate, k_{max}^i [mol CE mol bacteria^{-1} s^{-1}] is the maximum concentration of CE that can be degraded by a mole of *Dehalococcoides* per unit of time, and K_i [mol L^{-1}] is the half-saturation constant.

Table S2. List of calibrated kinetic parameter values used in this study, with the corresponding unit and the explicit reference to the type of model.

Parameter	Unit	Value	Kinetics*
a_{cDCE}^{tceA}	L mol CE^{-1} s^{-1}	1.5e0	E
a_{VC}^{tceA}	L mol CE^{-1} s^{-1}	1.3e-2	E
a_{cDCE}^{vcrA}	L mol CE^{-1} s^{-1}	1.5e-4	E
a_{VC}^{vcrA}	L mol CE^{-1} s^{-1}	8.5e-0	E
k_{dec}^{X}	s^{-1}	5.0e-4	E
β_{tceA}^{T}	mol transcripts mol biomass^{-1}	8.0e-2	E
β_{vcrA}^{T}	mol transcripts mol biomass^{-1}	3.0e-1	E
β_{TceA}^{E}	mol enzymes mol transcripts^{-1}	4.0e3	E
β_{VcrA}^{E}	mol enzymes mol transcripts^{-1}	1.0e3	E
k_{h}^{XtceA}	-	1.0e-1	E
k_{h}^{XvcrA}	-	1.0e-1	E
k_{max}^{cDCE}	mol CE mol enzymes^{-1} s^{-1}	3.3e-9	E
k_{max}^{VC}	mol CE mol enzymes^{-1} s^{-1}	7.8e-10	E
$k_{h,EBK}^{cDCE}$	mol L^{-1}	7.0e-6	E
$k_{h,EBK}^{VC}$	mol L^{-1}	1.0e-9	E
μ_{max}^{cDCE}	s^{-1}	1.7e-6	M
μ_{max}^{VC}	s^{-1}	7.0e-7	M
$k_{h,Monod}^{cDCE}$	mol L^{-1}	8.0e-6	M
$k_{h,Monod}^{VC}$	mol L^{-1}	9.0e-7	M
Y_{cDCE}	mol of biomass mol^{-1} of substrate	3.5	E,M
Y_{VC}	mol of biomass mol^{-1} of substrate	3.7	E,M
k_{dec}^{B}	s^{-1}	6.0e-9	E,M
k_{FO}^{Eth}	s^{-1}	2.1e-6	FO

* E = Enzyme-based Kinetics; M = Monod kinetics; FO = First-order kinetics.

Table S3. Relevant batch and 1D model features, such as initial and source concentration, and conversion factors.

Description	Unit	Value	Model
Initial *Dehalococcoides* concentration[a]	genes L^{-1} (genes mL^{-1})	3.0e8 (3.0e5)	Batch models
Initial cis-DCE concentration[a]	μmol L^{-1}	47.5	Batch models
Initial chloride concentration[a]	μmol L^{-1}	140.0	Batch models
Sedimentary organic carbon	%	0.5	1D flow model
Fraction of immobile *Dehalococcoides*[b]	%	85	1D flow model
Fraction of mobile *Dehalococcoides*[b]	%	15	1D flow model
Initial amount of immobile *Dehalococcoides*[c]	genes kg^{-1} (genes mass of soil containing 1 L^{1} of pore water)	9.2e2 (5.7e3)	1D flow model
Initial concentration of mobile *Dehalococcoides*[c]	genes L^{-1} (genes mL^{-1})	1.0e3 (1.0)	1D flow model
Source cis-DCE concentration	μmol L^{-1}	50.0	1D flow model
Maximum amount of *Dehalococcoides* in the pore space[d]	genes kg^{-1} (genes mass of soil containing 1 L^{-1} of pore water)	2.0e7 (1.2e8)	1D flow model
Initial *tceA* transcription factor content	-	0.0	All models
Initial *vcrA* transcription factor content	-	0.0	All models
[mol L^{-1}] to [genes-transcripts-enzymes L^{-1}] conversion factor for biomass[e]	genes mol of biomass^{-1}	1.5e14	All models

[a] Estimated from experimental data.

[b] Estimated from Cápiro et al. (2014) and Hnatko et al. (2020).

[c] Estimated from Ottosen et al. (2020).

[d] Estimated from Takeuchi et al. (2011).

[e] Estimated from Balkwill et al. (1988).

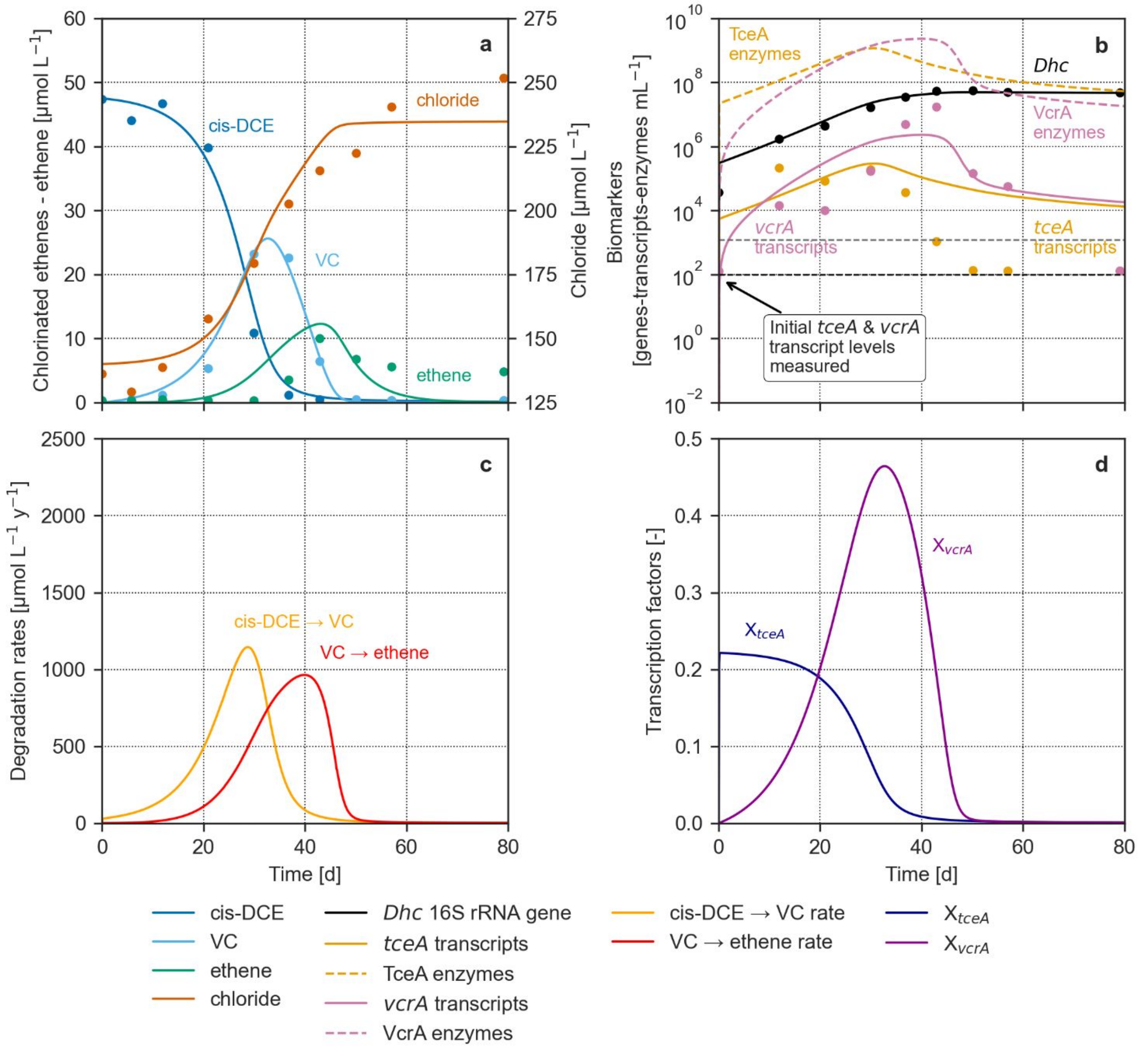

Figure S1. Temporal patterns (solid lines) of chemical concentrations (a), biomarker levels (b), degradation rates (c), and transcription factor (d) in the microcosm experiment in Kranzioch et al. (2015), obtained through the enzyme-based kinetics and considering both tceA and vcrA as non-homologous. In the plots, the concentration of 16S rRNA gene copies corresponds to the number of Dehalococcoides cells. The black and grey dashed lines represent the quantification limits of Dehalococcoides 16S rRNA gene and the tceA and vcrA transcripts, respectively. Refer to the original paper for the error bars related to the standard deviation of all the chemical and biomarker measurements.

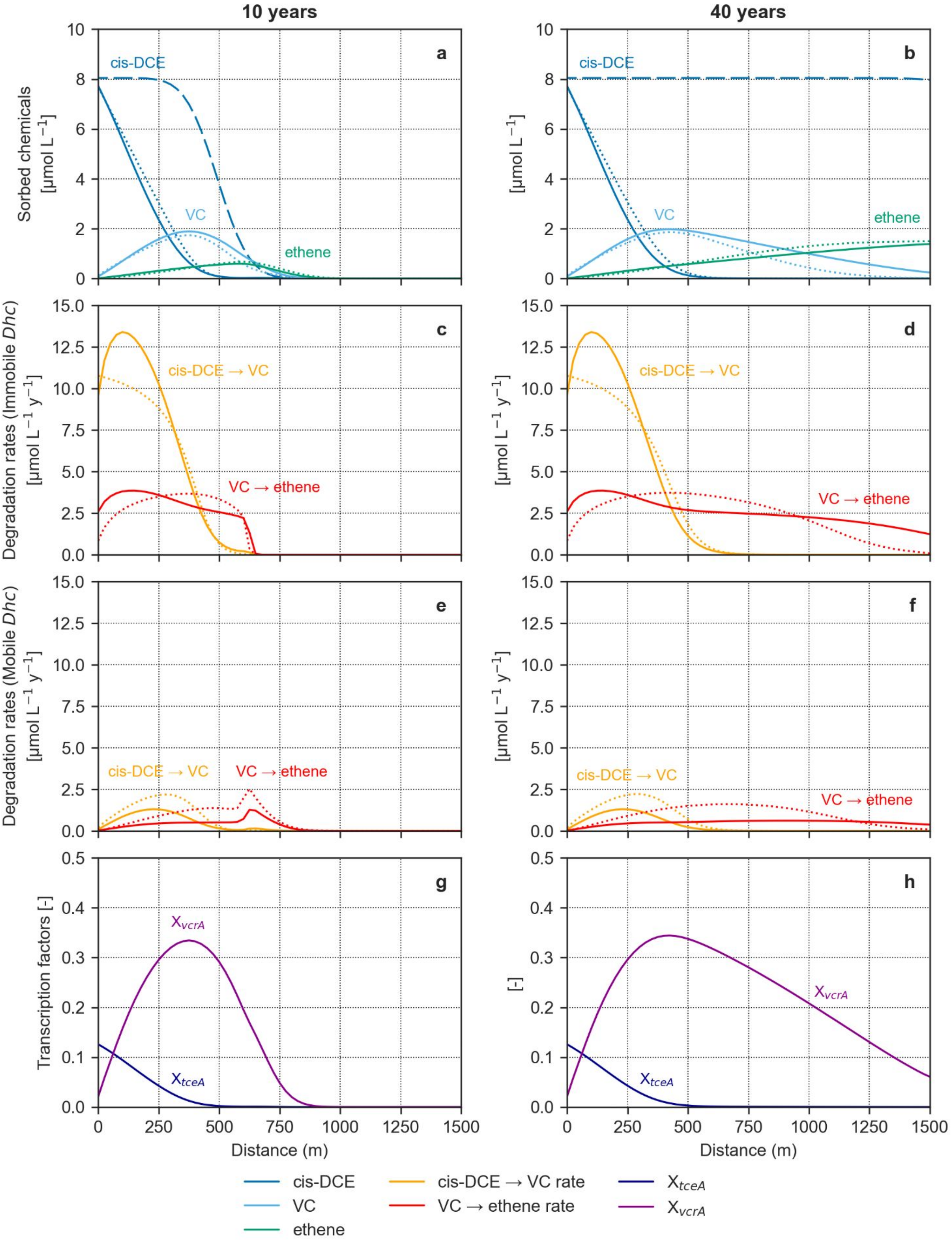

Figure S2. 1D scenario-based reactive transport model simulating two relevant stages of the evolution of a cis-DCE plume in groundwater: the elongation phase at 10 years, when the plume is in a transient state, and the steady-state condition at 40 years. In the plot, the sorbed chemicals (a,b), the degradation rates due to the immobile (c,d) and mobile (e,f) biomarkers, and the transcription factors (g,h). The solid lines refer to the enzyme-based 1D flow model, the dotted to the Monod-based, while the long-dashed lines ones to the conservative transport of cis-DCE. In the plots, the concentration of 16S rRNA gene copies corresponds to the number of Dehalococcoides cells.

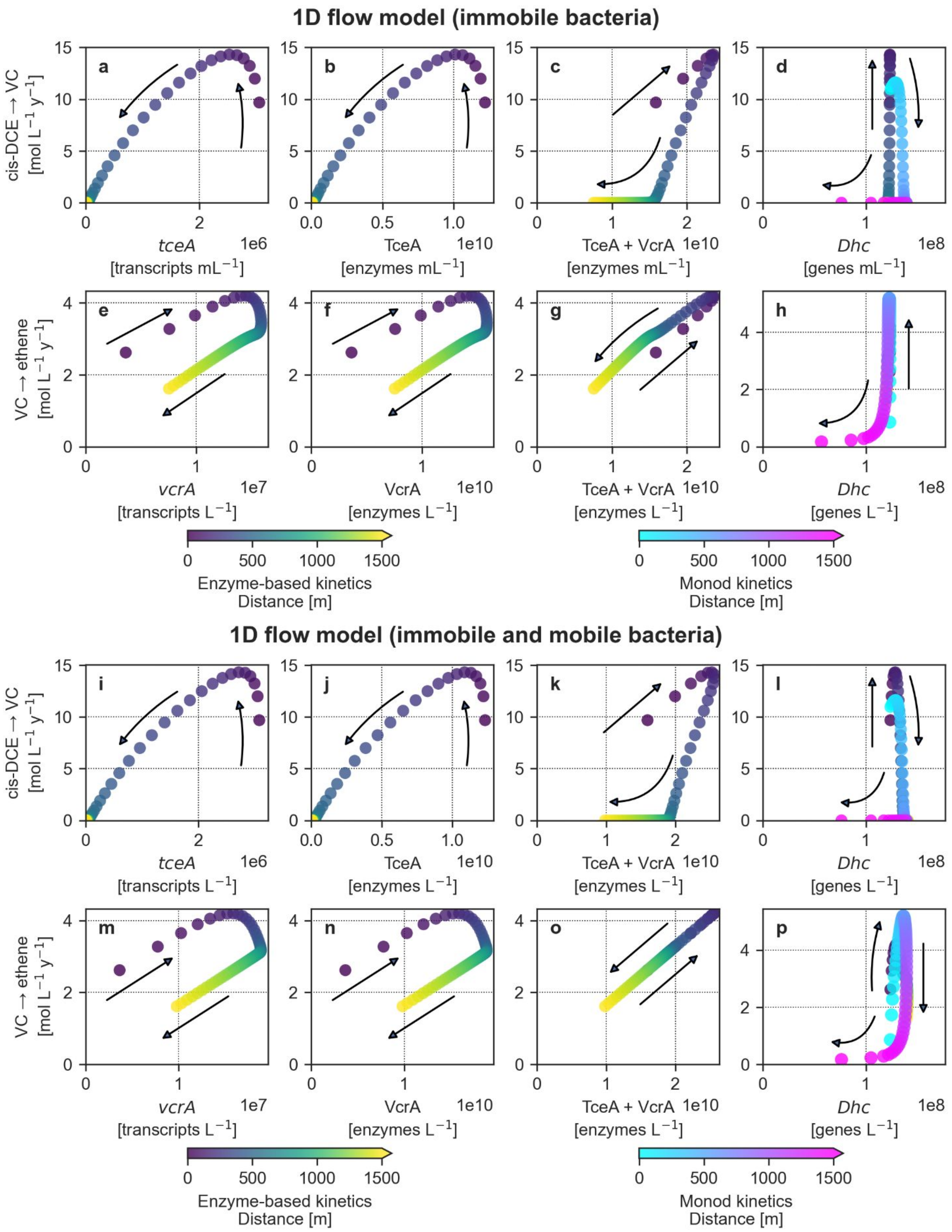

Figure S3. Relationships between biomarker levels and degradation rates of the two transformation steps of cis-DCE, via VC, to ethene, related to the 1D flow model, considering only immobile biomarkers (a-h) and the combination of immobile and mobile biomarkers (i-p). Each plot describes how the biomarker-rate relationship distance (color scales).

Table S4. Pearson correlation coefficients (r) of the relationships between the biomarker levels and degradation rates of the two transformation steps of cis-DCE, via VC, to ethene, referred to panels in Fig. 4 in the main text.

	**Batch model (EBK)**	**Batch model (Monod)**	**40-year 1D flow model (EBK)**	**40-year 1D flow model (Monod)**
cis-DCE → VC vs. *tceA* transcripts	0.986 (Fig. 4a)	-	0.932 (Fig. 4i)	-
cis-DCE → VC vs. TceA enzymes	0.986 (Fig. 4b)	-	0.932 (Fig. 4j)	-
cis-DCE → VC vs. TceA + VcrA enzymes	0.419 (Fig. 4c)	-	-0.560 (Fig. 4k)	-
cis-DCE → VC vs. *Dehalococcoides* spp.	-0.371 (Fig. 4d)	-0.417 (Fig. 4d)	-0.778 (Fig. 4l)	-0.787 (Fig. 4l)
VC → ethene vs. *vcrA* transcripts	0.986 (Fig. 4e)	-	-0.006 (Fig. 4m)	-
VC → ethene vs. VcrA enzymes	0.986 (Fig. 4f)	-	-0.006 (Fig. 4n)	-
VC → ethene vs. TceA + VcrA enzymes	0.999 (Fig. 4g)	-	0.204 (Fig. 4o)	-
VC → ethene vs. *Dehalococcoides* spp.	-0.026 (Fig. 4h)	0.003 (Fig. 4h)	-0.268 (Fig. 4p)	-0.111 (Fig. 4p)

Table S5. Hysteresis Loop Areas (HLAs) of the relationships between the biomarker levels and degradation rates of the two transformation steps of cis-DCE, via VC, to ethene, referred to panels in Fig. 4 in the main text. HLAs relate to the surface enclosed in hysteretic non-linear curves to measure the degree of hysteresis using the trapezoidal integration rule (Onoue, 2019). To remove the possible effect of the variability ranges of biomarker levels and degradation rates, each variable was previously scaled to the [0,1] range. In this way, the maximum reference area becomes equal to 1.

	**Batch model (EBK)**	**Batch model (Monod)**	**40-year 1D flow model (EBK)**	**40-year 1D flow model (Monod)**
cis-DCE → VC vs. *tceA* transcripts	0.164 (Fig. 4a)	-	0.427 (Fig. 4i)	-
cis-DCE → VC vs. TceA enzymes	0.163 (Fig. 4b)	-	0.427 (Fig. 4j)	-
cis-DCE → VC vs. TceA + VcrA enzymes	0.587 (Fig. 4c)	-	0.722 (Fig. 4k)	-
cis-DCE → VC vs. *Dehalococcoides* spp.	0.436 (Fig. 4d)	0.460 (Fig. 4d)	0.489 (Fig. 4l)	0.596 (Fig. 4l)
VC → ethene vs. *vcrA* transcripts	0.167 (Fig. 4e)	-	0.553 (Fig. 4m)	-
VC → ethene vs. VcrA enzymes	0.167 (Fig. 4f)	-	0.553 (Fig. 4n)	-
VC → ethene vs. TceA + VcrA enzymes	0.000 (Fig. 4g)	-	0.539 (Fig. 4o)	-
VC → ethene vs. *Dehalococcoides* spp.	0.686 (Fig. 4h)	0.569 (Fig. 4h)	0.618 (Fig. 4p)	0.709 (Fig. 4p)

References

1. Appelo, C. A. J., & Postma, D. (2004). Geochemistry, groundwater and pollution. CRC press.
2. Balkwill, D. L., Leach, F. R., Wilson, J. T., McNabb, J. F., & White, D. C. (1988). Equivalence of microbial biomass measures based on membrane lipid and cell wall components, adenosine triphosphate, and direct counts in subsurface aquifer sediments. Microbial Ecology, 16, 73-84.
3. Cápiro, N. L., Wang, Y., Hatt, J. K., Lebrón, C. A., Pennell, K. D., & Löffler, F. E. (2014). Distribution of organohalide-respiring bacteria between solid and aqueous phases. Environmental Science & Technology, 48(18), 10878-10887.
4. Clement, T. P., Peyton, B. M., Skeen, R. S., Jennings, D. A., & Petersen, J. N. (1997). Microbial growth and transport in porous media under denitrification conditions: experiments and simulations. Journal of Contaminant Hydrology, 24(3-4), 269-285.
5. Doherty, J. (2004). PEST: Model-Independent Parameter Estimation – User Manual: 5th Edition. Watermark Numerical Computing.
6. Hansch, C., Leo, A., & Hoekman, D. (1995). Exploring QSAR: hydrophobic, electronic, and steric constants (Vol. 2). Washington, DC: American Chemical Society.
7. Hill, M. C. (1998). Methods and guidelines for effective model calibration: with application to UCODE, a computer code for universal inverse modeling, and MODFLOWP, a computer code for inverse modeling with MODFLOW (Vol. 98, No. 4005). US Geological Survey.
8. Hnatko, J. P., Yang, L., Pennell, K. D., Abriola, L. M., & Cápiro, N. L. (2020). Bioenhanced back diffusion and population dynamics of Dehalococcoides mccartyi strains in heterogeneous porous media. Chemosphere, 254, 126842.
9. Karickhoff, S. W. (1981). Semi-empirical estimation of sorption of hydrophobic pollutants on natural sediments and soils. Chemosphere, 10(8), 833-846.

10. Kranzioch, I., Ganz, S., & Tiehm, A. (2015). Chloroethene degradation and expression of Dehalococcoides dehalogenase genes in cultures originating from Yangtze sediments. Environmental Science and Pollution Research, 22(4), 3138-3148.
11. Kranzioch, I., Stoll, C., Holbach, A., Chen, H., Wang, L., Zheng, B., Norra, S., Bi, Y., Schramm, K.W., & Tiehm, A. (2013). Dechlorination and organohalide-respiring bacteria dynamics in sediment samples of the Yangtze Three Gorges Reservoir. Environmental Science and Pollution Research, 20, 7046-7056.
12. Onoue, K. (2019). Energy consumption characteristics of concrete using granulated blast-furnace slag sand related to nucleation and propagation of microcracks. Construction and Building Materials, 218, 404-412.
13. Ottosen, C. B., Rønde, V., McKnight, U. S., Annable, M. D., Broholm, M. M., Devlin, J. F., & Bjerg, P. L. (2020). Natural attenuation of a chlorinated ethene plume discharging to a stream: Integrated assessment of hydrogeological, chemical and microbial interactions. Water Research, 186, 116332.
14. Phanikumar, M. S., Hyndman, D. W., Zhao, X., & Dybas, M. J. (2005). A three-dimensional model of microbial transport and biodegradation at the Schoolcraft, Michigan, site. Water Resources Research, 41(5).
15. Ran, Y., He, Y., Yang, G., Johnson, J. L., & Yalkowsky, S. H. (2002). Estimation of aqueous solubility of organic compounds by using the general solubility equation. Chemosphere, 48(5), 487-509.
16. Sakuratani, Y., Kasai, K., Noguchi, Y., & Yamada, J. (2007). Comparison of predictivities of log P calculation models based on experimental data for 134 simple organic compounds. QSAR & Combinatorial Science, 26(1), 109-116.
17. Schaefer, C. E., Condee, C. W., Vainberg, S., & Steffan, R. J. (2009). Bioaugmentation for chlorinated ethenes using Dehalococcoides sp.: Comparison between batch and column experiments. Chemosphere, 75(2), 141-148.

18. Takeuchi, M., Kawabe, Y., Watanabe, E., Oiwa, T., Takahashi, M., Nanba, K., ... & Komai, T. (2011). Comparative study of microbial dechlorination of chlorinated ethenes in an aquifer and a clayey aquitard. Journal of contaminant hydrology, 124(1-4), 14-24.
